# Effects of Dance and Rhythmic Physical Activity Interventions on Executive Function and Motor Competence in Children: A Systematic Review and Three-Level Meta-Analysis

**DOI:** 10.3390/bs16091699

**Published:** 2026-09-20

**Authors:** Hao Luo, Lingyun Long, Haozhe Wang, Wenjia Chen

**Affiliations:** 1 Department of Physical Education, Chongqing Electric Power College, Chongqing 400053, China; luohaaaa@gmail.com; 2 Department of Education, General Graduate School, Soonchunhyang University, Asan 31538, Republic of Korea; longlingyun2026@163.com; 3 School of Physical Education, China University of Mining and Technology, Xuzhou 221116, China; ts24110007a31@cumt.edu.cn

**Keywords:** dance, rhythmic physical activity, children, executive function, motor competence, three-level meta-analysis

## Abstract

Dance and rhythmic physical activity may support motor and cognitive development in children, but the magnitude and consistency of these effects remain uncertain, and individual trials frequently contribute multiple statistically dependent outcomes. Five electronic databases and ClinicalTrials.gov were searched from inception to May 2026. Executive function (EF) and motor competence (MC) were analyzed as separate primary domains using multilevel random-effects models with an estimated sampling variance–covariance structure and trial-clustered CR2 robust inference. Cluster-randomized designs, shared controls, and overlapping reports were explicitly accounted for. Twenty-two reports representing 21 unique trials and 2496 unique children were included. The primary EF model included 34 core-EF effect sizes from seven trials and yielded g = 0.50 (95% CI [−0.04, 1.04], *p* = 0.063; 95% PI [−1.24, 2.24]). The MC model included 103 effect sizes from 17 trials and showed a positive average effect (g = 0.95, 95% CI [0.39, 1.52], *p* = 0.003; 95% PI [−1.43, 3.34]). Trial-clustered dose–response models did not identify statistically supported associations for intervention duration, total minutes, weekly frequency, or session duration. Certainty of evidence was very low for EF and low for MC. Dance and rhythmic physical activity may improve motor competence in children on average, although effects vary substantially across settings. Executive-function findings remain preliminary and statistically uncertain. Current evidence does not support causal recommendations for longer interventions or higher total doses.

## 1. Introduction

Childhood is a critical period for the development of executive function and motor competence. Executive function reflects an individual’s capacity to regulate attention, memory, and responses during goal-directed behavior and constitutes an important cognitive foundation for completing learning tasks, controlling behavioral responses, and adapting to school and daily life (Best & Miller, 2010; Diamond, 2013; Zelazo & Carlson, 2012). Motor competence reflects children’s ability to perform fundamental movements and engage in physical activity, and its development influences current participation and may contribute to later physical activity trajectories (Barnett et al., 2022; Robinson et al., 2015; Stodden et al., 2008). Executive function and motor competence are also related during development: better motor competence may support participation in more complex physical activities, whereas cognitively demanding movement tasks require motor planning, rule switching, and sustained control (Bao et al., 2024; de Greeff et al., 2018; Mao et al., 2024). Examining interventions that combine motor coordination with cognitive engagement may therefore help clarify how movement experiences support children’s motor and cognitive development.

Dance and rhythmic physical activity provide a suitable intervention context for this question. Rather than merely increasing activity time, these activities require continuous bodily control in response to rhythmic cues and movement rules (Bläsing et al., 2012; Karpati et al., 2015; Teixeira-Machado et al., 2019). Learning and adjusting movement sequences requires children to translate external beats into coordinated actions, remember movement patterns, and modify responses when the sequence or rhythm changes (Chen et al., 2008; Repp & Su, 2013; Thaut et al., 2014). Thus, the relevant contrast is not simply dance versus exercise but activities with structured rhythmic cueing, motor sequencing, and coordination demands versus activities with less variation in these cognitive–motor requirements. These characteristics provide plausible pathways through which rhythmic movement may influence both executive function and motor competence.

Randomized trials have examined DanceSport, creative dance, digital dance games, action–song programs, aerobic/fitness dance, and music- or rhythm-based movement (Bentley et al., 2023; W. Liu et al., 2025; Oppici et al., 2020; van den Berg et al., 2019). Although these interventions differ in format, the interventions considered in this review share three central features: structured whole-body movement, external rhythmic or musical cueing as an organizing element, and repeated sequencing or coordination demands. Relevant reviews have synthesized dance, physical activity, motor competence, and executive-function outcomes from broader perspectives (Fong Yan et al., 2018, 2024; González-del-Castillo & Barbero-Alcocer, 2025; González-Devesa et al., 2026; C. Liu et al., 2025; Richter et al., 2025; Tao et al., 2025; Vazou et al., 2019; Wang et al., 2024; Yang et al., 2025), but the statistical dependence created when individual trials report multiple outcomes remains an important synthesis problem. A review focused on randomized evidence and on these two movement-science domains can therefore provide a more specific estimate while retaining the information contained in multiple outcomes from the same trial.

Accordingly, this study aimed to evaluate the effects of dance and rhythmic physical activity on executive function and motor competence in children, with the two domains analyzed separately as primary outcomes. A three-level/multivariate random-effects framework was used to retain multiple outcomes while accounting for their dependence. Intervention dose variables were examined because the duration and amount of repeated practice may plausibly influence motor learning and cognitive transfer; however, given the limited number of independent trials, moderator and dose–response analyses were treated as exploratory rather than confirmatory.

## 2. Methods

### 2.1. Protocol and Reporting Guidelines

This systematic review and three-level meta-analysis was reported in accordance with the PRISMA 2020 statement (Page et al., 2021), and the completed checklist is provided as a separate Supplementary File. The protocol was registered in PROSPERO in January 2026 (CRD420261445431), before title/abstract screening and statistical analysis. The registered eligibility criteria and outcome domains were retained. During peer-review revision, the statistical analysis was refined to treat executive function and motor competence as separate primary syntheses, restrict the primary executive-function construct to core domains, and more explicitly account for overlapping reports, cluster randomization, shared controls, correlated sampling errors, and small-sample inference; these refinements did not alter study eligibility.

### 2.2. Eligibility Criteria

Studies were eligible if participants were children or adolescents aged 3–18 years; the intervention was a structured dance or rhythmic physical activity lasting at least 4 weeks; the comparator was regular physical education, usual curriculum, wait-list/no intervention, health education, or another physical activity; at least one eligible executive-function or motor-competence outcome was reported with sufficient quantitative data; and the design was randomized, including individually randomized and cluster-randomized trials. Eligibility was operationalized around three intervention features: structured whole-body movement, rhythmic or musical cueing that organized the activity, and repeated motor sequencing/coordination as a central active component. Digital dance games, action–song programs, and movement-to-music interventions were eligible only when these features were substantial rather than incidental. Multicomponent programs were included only when the dance/rhythm component was the dominant or separately identifiable active component. The 4-week minimum was chosen to distinguish repeated training from acute or very short exposure and to allow time for motor learning or adaptation to occur.

Studies were excluded if participants were adults, older adults, or professional/elite dancers; samples were primarily clinical rehabilitation populations or individuals with severe neurodevelopmental disorders; the intervention consisted of a single acute session, music therapy without structured physical movement, psychological therapy alone, or a multicomponent program from which the rhythmic-movement component could not be isolated; or the study lacked random allocation, a comparator, or extractable quantitative outcomes. No language-based exclusion criterion was specified; publication status was not restricted to journal articles, and dissertations/theses and registered trials were eligible when sufficient information was available. Although the original eligibility range extended to 18 years, no eligible trial ultimately included an adolescent sample aged 13–18 years, and the title and interpretation were therefore restricted to children.

### 2.3. Data Sources and Search Strategy

PubMed/MEDLINE, Web of Science, Scopus, ERIC, and ProQuest Dissertations and Theses Global were systematically searched, and ClinicalTrials.gov was searched as a trial registry. The search covered the period from database inception to May 2026.

The search strategy was developed around three concepts: dance or rhythmic physical activity, children/youth, and executive function or motor competence. Search strategies were adapted to the controlled vocabulary and free-text conventions of each source. The complete search strategies are provided in the Supplementary Materials.

### 2.4. Study Selection and Data Extraction

Search records were imported into EndNote X9 for deduplication. Two reviewers independently screened titles/abstracts and full texts against the prespecified criteria; disagreements were resolved by discussion and, when necessary, by a third reviewer. Reasons for full-text exclusion were recorded for the PRISMA flow diagram. During the final trial audit, reports were also linked to underlying trials so that multiple publications from the same participant cohort were not counted as independent studies.

Two reviewers independently extracted data using the same prespecified standardized form and compared entries item by item before analysis; no formal kappa statistic was calculated because most extraction fields were continuous. Extracted variables included study and sample characteristics, intervention and comparator conditions, randomization unit, intervention arms and shared controls, outcome measures, follow-up timing, data required for effect-size calculation, and risk-of-bias information. All primary effects could be reconstructed from the published reports, so no unpublished author-supplied data were used. No graphically digitized value was required for the final primary analyses. Where statistics required conversion, the calculation and source were recorded and independently checked against the original report.

### 2.5. Outcomes and Coding of Variables

The primary outcomes were executive function and motor competence. The primary executive-function construct was restricted to the three core domains of inhibitory control, working memory, and cognitive flexibility, together with composite scores predominantly reflecting these domains (Diamond, 2013). Measures of selective/alerting/orienting attention, semantic fluency, and planning/problem solving were considered conceptually broader and were excluded from the primary EF model but retained in a broad-EF sensitivity analysis. Motor competence included gross motor skills, balance, coordination, locomotor skills, object control, fine/visual-motor competence, and standardized motor-proficiency scores. Outcomes for which lower values indicated better performance, such as reaction time, error rate, or postural sway, were reversed so that positive Hedges’ g values consistently favored the intervention.

Intervention type, participant age, comparator condition, intervention duration, weekly frequency, session duration, and total intervention minutes were extracted according to prespecified coding rules. Because only 7 independent trials contributed to the primary EF model and 17 to the MC model, a large set of categorical moderators was not treated as confirmatory tests. Prespecified categorical subgroup information was retained descriptively, whereas formal meta-regression focused on a small number of exploratory dose variables and used small-sample robust inference.

### 2.6. Risk of Bias and Certainty of Evidence

Risk of bias was reassessed at the result level using the Cochrane Risk of Bias 2 (RoB 2) tool, targeting the effect of assignment to intervention. The standard RoB 2 tool was applied to individually randomized parallel-group trials, whereas the cluster-randomized version was used for cluster trials, including the additional domain concerning identification and recruitment of participants within clusters. When multiple outcomes within the same outcome family shared the same measurement and analytic features, a common result-level judgement was applied; outcomes with materially different measurement processes were assessed separately. Two reviewers independently completed the assessments, with disagreements resolved by discussion and, when necessary, third-reviewer adjudication. Judgements followed the RoB 2 algorithms and were classified as low risk, some concerns, or high risk.

Certainty of evidence was reassessed separately for executive function and motor competence using GRADE across risk of bias, inconsistency, indirectness, imprecision, and publication bias. Judgements incorporated the result-level RoB 2 assessments, CR2-robust confidence intervals, prediction intervals, influence analyses, and the number of independent trials.

### 2.7. Effect Size Calculation

Hedges’ g was used as the standardized effect measure with small-sample correction. For outcomes reported as arm-level pre- and post-intervention scores, the primary contrast was the between-group difference in change from baseline, standardized by the pooled change-score SD. When the change-score SD was not reported, it was reconstructed as SDchange = sqrt(SDpre^2 + SDpost^2 − 2 × r × SDpre × SDpost), using r = 0.50 in the primary analysis and r = 0.20 and 0.80 in sensitivity analyses. Source-reported change-score precision was used directly when available. Conventional standardized-mean-difference benchmarks were used only as descriptive context (Cohen, 1988); interpretation emphasized the point estimate, 95% CI, and 95% prediction interval rather than fixed labels.

All effects were aligned so that positive values indicated better outcomes in the intervention group. For lower-is-better outcomes, the sign was reversed before synthesis. When an eligible study reported an adjusted model estimate rather than directly usable arm-level change data, the estimate was converted using the most appropriate reported standardizer rather than treating a model standard error as an outcome SD. In particular, van den Berg et al. (2019) mixed-model coefficients were standardized using pooled baseline SDs, while the immediate post-intervention contrast was used for Williams et al. (2026), and the source-adjusted longitudinal estimate was examined in sensitivity analysis. For Gencigör and Akın (2024), group-by-time F statistics were converted to the corresponding standard error/change-score precision. Study-specific conversions are documented in the Supplementary Materials.

The primary synthesis used the immediate post-intervention time point; later follow-up measurements were not entered simultaneously with immediate outcomes. Cluster-randomized trials based on raw arm data were adjusted using the design effect, DE = 1 + (m − 1)ICC, where m is the average cluster size. When an ICC was unavailable, ICC = 0.05 was used in the primary analysis with 0.01 and 0.10 examined in sensitivity analyses; a study-reported design effect was retained when available. If the original study provided an appropriately cluster-adjusted model estimate used directly in the meta-analysis, an additional design-effect correction was not applied. No true crossover sequence with treatment switching contributed to the final synthesis; the report labeled as crossover used a parallel-group data structure and was analyzed accordingly. Effect-size conversions followed standard meta-analytic guidance (Higgins et al., 2024).

### 2.8. Statistical Analysis

Executive function and motor competence were analyzed as separate primary domains. Sampling variances and covariances were assembled in a working variance–covariance matrix so that multiple outcomes from the same participant cohort, shared-control comparisons, and other correlated sampling errors were represented rather than assumed independent. A working within-cohort correlation of rho = 0.50 was used, with rho = 0.20 and 0.80 examined in sensitivity analyses. Jouira et al. (2024) and Zinelabidine et al. (2022) were treated as two reports from one underlying randomized trial and assigned the same trial identifier. Multi-arm comparisons sharing a control group were retained within the same covariance structure.

For each domain, a multilevel random-effects model was fitted by restricted maximum likelihood, with effect sizes nested within the underlying trial. Inference for pooled effects and meta-regression coefficients used trial-clustered CR2 robust variance estimation with small-sample Satterthwaite degrees of freedom. Hedges’ g, 95% CI, and 95% prediction interval were reported, and heterogeneity was partitioned into within-trial and between-trial components. Analyses were implemented in R 4.5.2 using metafor and clubSandwich (Pustejovsky & Tipton, 2018; Viechtbauer, 2010). No combined executive-function plus motor-competence summary estimate was treated as a primary effect.

The prespecified moderator set was substantially reduced because the number of independent trials was small relative to the number of candidate moderators and several categorical cells were sparse. Formal conclusions were not based on whether separate subgroup estimates were individually significant. Any retained subgroup summaries were considered exploratory, and the main inferential emphasis remained on the two domain-specific models and the dose meta-regressions.

Dose–response analyses were conducted after dependency-aware aggregation to the Trial × Domain level so that studies reporting many outcomes did not receive disproportionate weight. Intervention duration, total minutes, weekly frequency, and session duration were examined in separate linear meta-regression models adjusted for outcome domain, with comparator type added in sensitivity models. Trial-clustered CR2 inference was used throughout. Nonlinear spline analyses were removed because the number and distribution of independent trials were insufficient for stable estimation. The four dose models were treated as exploratory, and multiplicity was considered using Holm adjustment.

Small-study effects were assessed only when the number of independent trials was sufficient. No formal Egger-type test was conducted for executive function because only seven trials contributed. For motor competence, dependent effects were first aggregated to one covariance-aware trial-level estimate, and an Egger-type regression was then fitted across the 17 independent trials. Funnel asymmetry was interpreted as a small-study-effect signal rather than direct proof of publication bias.

Sensitivity analyses examined the main assumptions rather than deleting effects according to an arbitrary magnitude threshold. Analyses varied the pre-post correlation (r = 0.20, 0.50, 0.80), the working sampling correlation (rho = 0.20, 0.50, 0.80), and cluster ICC assumptions (0.01, 0.05, 0.10); repeated the EF synthesis with the broader conceptual set of 41 effects; examined study-specific effect-size conversions and source-data issues; and used trial-level leave-one-out, Cook’s distance, DFBETAS, leverage, and standardized residuals to identify influential trials. Influential trials were retained in the primary model and excluded only in sensitivity refits.

## 3. Results

### 3.1. Study Selection

**Figure 1:**
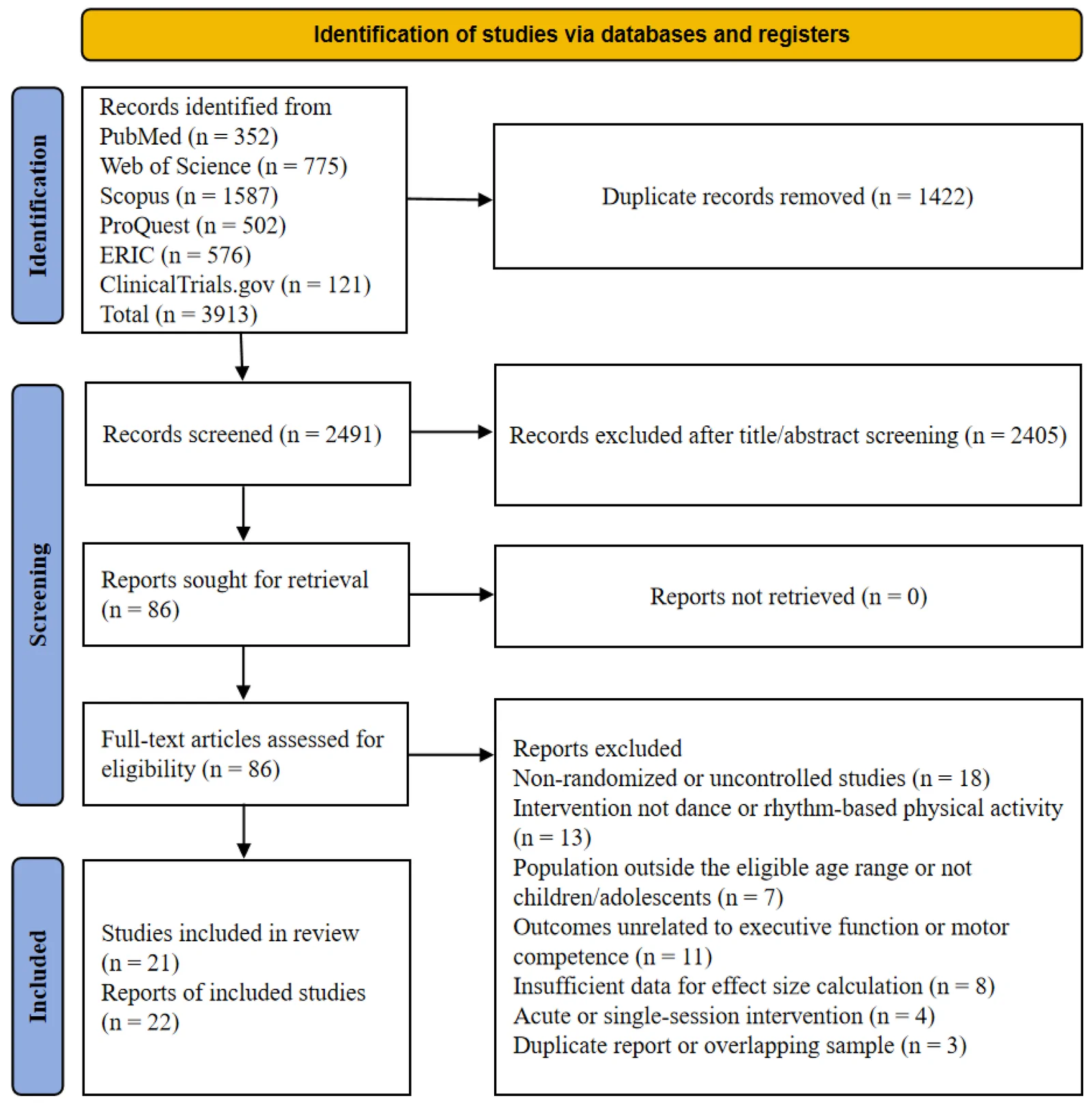
PRISMA 2020 flow diagram of study selection.

The database and trial-registry searches identified 3913 records: 352 from PubMed/MEDLINE, 775 from Web of Science, 1587 from Scopus, 502 from ProQuest Dissertations and Theses Global, 576 from ERIC, and 121 from ClinicalTrials.gov. After removing 1422 duplicates, 2491 records underwent title/abstract screening, and 86 reports were assessed in full text. Sixty-four full-text reports were excluded for ineligible design, intervention, population, outcome, insufficient quantitative data, acute/single-session exposure, or duplicate/overlapping reporting without unique eligible outcomes. Twenty-two reports representing 21 underlying trials were included (Anjos & Ferraro, 2018; Bentley et al., 2023; Chatzihidiroglou et al., 2018; Chatzipanteli et al., 2007; Chatzopoulos et al., 2022; Dalala et al., 2024; Derri et al., 2001; Đošić et al., 2025; Gencigör & Akın, 2024; Hu et al., 2020; Intawachirarat et al., 2026; Jouira et al., 2024; Kapodistria et al., 2021; W. Liu et al., 2025; Oppici et al., 2020; Tiktampanidi et al., 2021; van den Berg et al., 2019; Venetsanou et al., 2014; Williams et al., 2026; Zachopoulou et al., 2004; Zhao et al., 2024; Zinelabidine et al., 2022). Jouira et al. (2024) and Zinelabidine et al. (2022) reported different outcome domains from the same 41-child randomized trial and were therefore linked to one trial identifier. The selection process is shown in Figure 1.

### 3.2. Characteristics of Included Studies

The 22 included reports represented 21 unique trials and 2496 unique participants after the overlapping Jouira/Zinelabidine sample was counted once (Anjos & Ferraro, 2018; Bentley et al., 2023; Chatzihidiroglou et al., 2018; Chatzipanteli et al., 2007; Chatzopoulos et al., 2022; Dalala et al., 2024; Derri et al., 2001; Đošić et al., 2025; Gencigör & Akın, 2024; Hu et al., 2020; Intawachirarat et al., 2026; Jouira et al., 2024; Kapodistria et al., 2021; W. Liu et al., 2025; Oppici et al., 2020; Tiktampanidi et al., 2021; van den Berg et al., 2019; Venetsanou et al., 2014; Williams et al., 2026; Zachopoulou et al., 2004; Zhao et al., 2024; Zinelabidine et al., 2022). The characteristics of the included reports and underlying trials are summarized in Table 1. Individual report sample sizes ranged from 35 to 512 participants (Intawachirarat et al., 2026; van den Berg et al., 2019). Participants were approximately 3.35 to 12 years of age and were predominantly preschool- or primary-school children; no eligible trial included an adolescent sample aged 13–18 years. Most participants were typically developing children attending mainstream schools or kindergartens, although several studies focused on low physical activity, obesity, low socioeconomic status, or disadvantaged communities (Bentley et al., 2023; Intawachirarat et al., 2026; Jouira et al., 2024; Williams et al., 2026; Zinelabidine et al., 2022).

Interventions included creative dance, music-and-movement programs, rhythmic physical activity, DanceSport, fitness/aerobic dance, traditional dance, digital dance games, and other structured rhythm-based movement (Anjos & Ferraro, 2018; Bentley et al., 2023; Chatzihidiroglou et al., 2018; Chatzipanteli et al., 2007; Chatzopoulos et al., 2022; Dalala et al., 2024; Derri et al., 2001; Đošić et al., 2025; Gencigör & Akın, 2024; Hu et al., 2020; Intawachirarat et al., 2026; Jouira et al., 2024; Kapodistria et al., 2021; W. Liu et al., 2025; Oppici et al., 2020; Tiktampanidi et al., 2021; van den Berg et al., 2019; Venetsanou et al., 2014; Williams et al., 2026; Zachopoulou et al., 2004; Zhao et al., 2024; Zinelabidine et al., 2022). Intervention duration ranged from 4 weeks to one academic year, typically with 2–5 sessions per week lasting approximately 10–60 min (Anjos & Ferraro, 2018; Hu et al., 2020; Intawachirarat et al., 2026; Kapodistria et al., 2021; van den Berg et al., 2019). Seven independent trials contributed executive-function outcomes and 17 contributed motor-competence outcomes; three underlying trials contributed evidence to both domains. The included trials varied in intervention format, dose, comparator, and measurement approach, supporting the use of random-effects models while also requiring cautious interpretation of heterogeneity.

### 3.3. Risk of Bias

**Figure 2:**
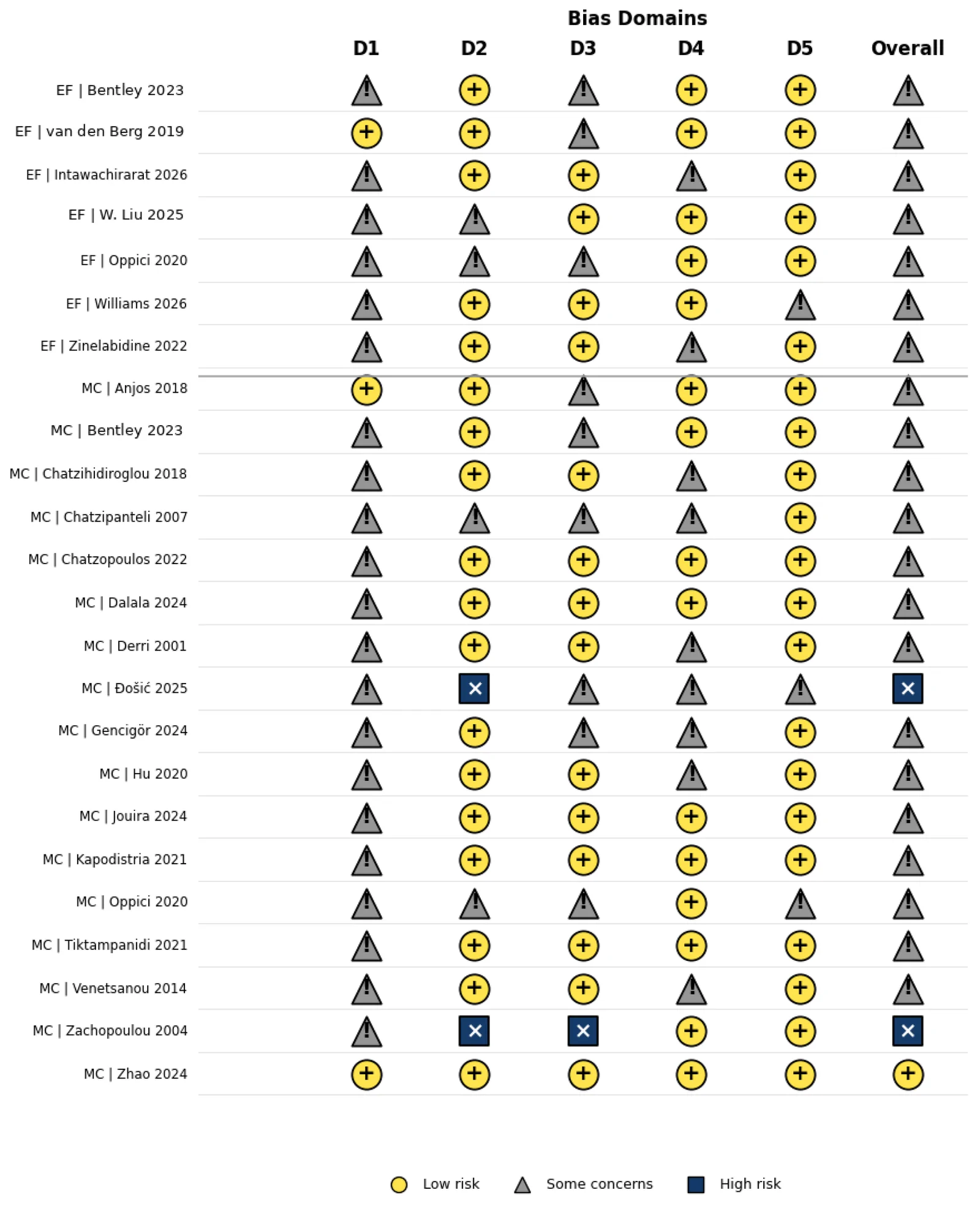
Result-level risk-of-bias judgments for executive-function and motor-competence outcomes. Author–Year labels identify the included reports (Anjos & Ferraro, 2018; Bentley et al., 2023; Chatzihidiroglou et al., 2018; Chatzipanteli et al., 2007; Chatzopoulos et al., 2022; Dalala et al., 2024; Derri et al., 2001; Đošić et al., 2025; Gencigör & Akın, 2024; Hu et al., 2020; Intawachirarat et al., 2026; Jouira et al., 2024; Kapodistria et al., 2021; W. Liu et al., 2025; Oppici et al., 2020; Tiktampanidi et al., 2021; van den Berg et al., 2019; Venetsanou et al., 2014; Williams et al., 2026; Zachopoulou et al., 2004; Zhao et al., 2024; Zinelabidine et al., 2022).

**Figure 3:**
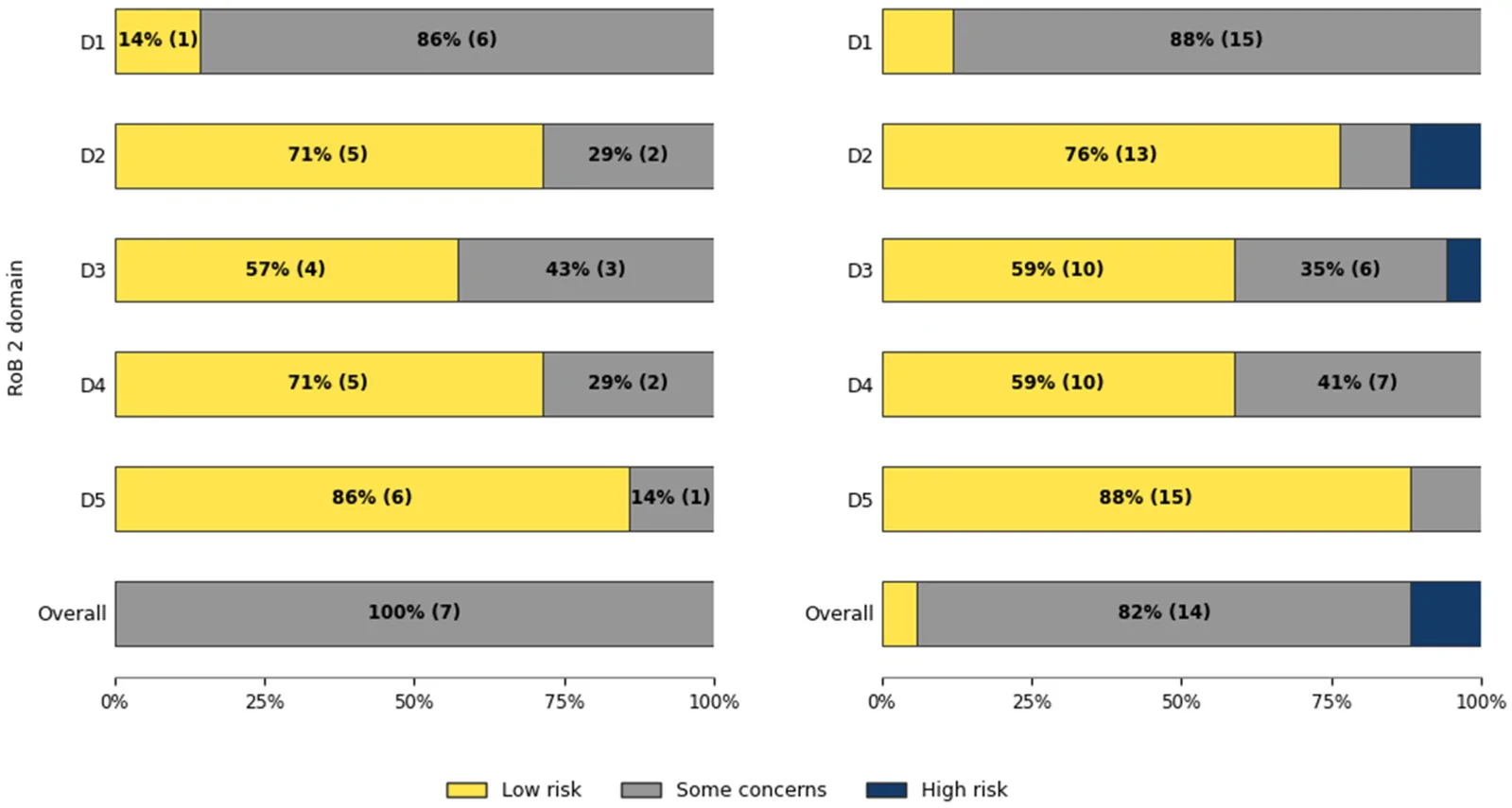
Distribution of result-level RoB 2 judgments across domains, shown separately for executive function and motor competence.

The RoB 2 assessments were conducted at the result/outcome-family level and are presented in Figure 2 and Figure 3 (Anjos & Ferraro, 2018; Bentley et al., 2023; Chatzihidiroglou et al., 2018; Chatzipanteli et al., 2007; Chatzopoulos et al., 2022; Dalala et al., 2024; Derri et al., 2001; Đošić et al., 2025; Gencigör & Akın, 2024; Hu et al., 2020; Intawachirarat et al., 2026; Jouira et al., 2024; Kapodistria et al., 2021; W. Liu et al., 2025; Oppici et al., 2020; Tiktampanidi et al., 2021; van den Berg et al., 2019; Venetsanou et al., 2014; Williams et al., 2026; Zachopoulou et al., 2004; Zhao et al., 2024; Zinelabidine et al., 2022). For executive function, all seven trial-level result assessments were judged as having some concerns, most commonly because allocation concealment, missing-data handling, or assessor blinding was incompletely reported.

For motor competence, 1 of 17 trial-level assessments (5.9%) was rated low risk, 14 (82.4%) had some concerns, and 2 (11.8%) were rated high risk. The two high-risk judgements were Đošić et al. (2025) and Zachopoulou et al. (2004), where post-randomization exclusions based on intervention attendance/participation were not compatible with the target effect of assignment to intervention.

Across outcome families, the most frequent concerns involved incomplete reporting of randomization or allocation concealment, post-randomization missing data, and assessor blinding for performance-based outcomes. Cluster-randomized trials were additionally judged for the timing and process of participant identification/recruitment relative to cluster allocation. These result-level judgements were carried forward into the GRADE assessment.

### 3.4. Domain-Specific Effects

**Figure 4:**
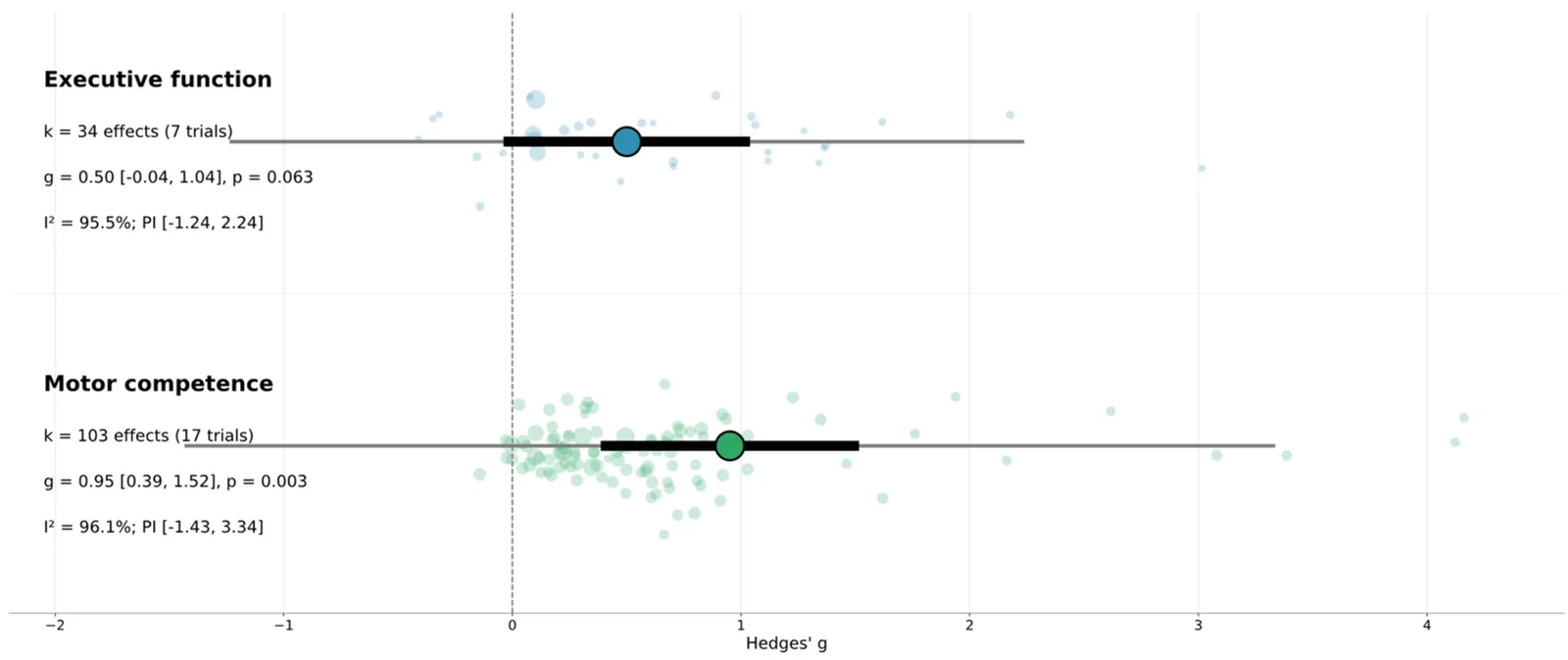
Three-level meta-analysis of the two primary outcome domains. Points show pooled Hedges’ g, thick lines show 95% confidence intervals, and thin lines show 95% prediction intervals. Inference uses trial-clustered CR2 robust standard errors.

Because executive function and motor competence represent distinct constructs, they were synthesized separately rather than combined into a single primary summary. The primary EF model included 34 core-EF effect sizes from 7 independent trials and yielded a positive but statistically uncertain average estimate (Hedges’ g = 0.50, 95% CI [−0.04, 1.04], *p* = 0.063; 95% PI [−1.24, 2.24]; Figure 4). The primary MC model included 103 effect sizes from 17 independent trials and showed a positive average effect (g = 0.95, 95% CI [0.39, 1.52], *p* = 0.003; 95% PI [−1.43, 3.34]; Figure 4). Both prediction intervals crossed zero, indicating substantial variability in the effects expected across future settings.

Heterogeneity remained substantial after explicitly accounting for sampling dependence and cluster design. For executive function, total I^2^ was 95.5%, with 47.5% attributable to between-trial heterogeneity and 48.0% to within-trial heterogeneity. For motor competence, total I^2^ was 96.1%, with 90.4% attributable to between-trial heterogeneity and 5.7% to within-trial heterogeneity. Thus, the positive MC average should not be interpreted as a uniform effect across settings, and the EF estimate remained particularly uncertain because it was based on only seven independent trials.

The domain-specific results therefore differed in evidential strength: the motor-competence estimate remained statistically supported under small-sample robust inference, whereas the executive-function confidence interval included the null. Subsequent analyses were interpreted in relation to this difference rather than to a combined overall effect.

### 3.5. Moderator and Subgroup Analyses

The originally prespecified categorical moderator set was not used for confirmatory inference after the trial-level audit because the number of independent trials was limited and several categories were represented by few trials. In particular, no formal study-level moderator model was emphasized for executive function, for which only seven trials were available. Descriptive subgroup summaries were retained in the Supplementary Materials but were not interpreted by comparing whether separate within-subgroup *p* values crossed the conventional significance threshold.

For motor competence, categorical subgroup findings were likewise treated as exploratory. The formal meta-regression component therefore focused on intervention dose variables, for which the number of contributing trials could be stated explicitly and small-sample CR2 inference could be applied (Section 3.8).

### 3.6. Sensitivity Analyses

Sensitivity analyses showed that the domain-level inference was stable to the main dependency assumptions. With pre-post correlations of r = 0.20, 0.50, and 0.80, EF estimates were g = 0.43, 0.50, and 0.69, respectively, and all robust confidence intervals crossed zero; corresponding MC estimates were g = 0.81, 0.95, and 1.30, with all confidence intervals excluding zero. Varying the working within-cohort sampling correlation (rho = 0.20–0.80) or assumed cluster ICC (0.01–0.10) likewise did not change the inference in either domain.

Conceptual and influence analyses reinforced the greater uncertainty for EF. Reintroducing the seven broader EF indicators produced g = 0.46 (95% CI [−0.06, 0.99], *p* = 0.073), while a change-score-only EF analysis produced g = 0.63 (95% CI [−0.15, 1.40], *p* = 0.087). W. Liu et al. (2025) was the most influential EF trial; excluding it reduced the estimate to g = 0.31 (95% CI [−0.12, 0.75], *p* = 0.122). In the MC model, Venetsanou et al. (2014) and Gencigör and Akın (2024) were the most influential trials, but removing either retained a positive statistically supported average effect (g = 0.77 and 0.78, respectively; both *p* < 0.01). Excluding the Gencigör effects reconstructed from F statistics yielded g = 0.78 (95% CI [0.31, 1.25], *p* = 0.003). No effect was excluded solely because its absolute Hedges’ g exceeded a preset threshold.

### 3.7. Publication Bias and Small-Study Effects

A formal small-study-effect test was not conducted for executive function because only seven independent trials contributed, making such regression unstable. For motor competence, the 103 dependent effects were first reduced to 17 covariance-aware trial-level estimates before the Egger-type regression. This analysis indicated trial-level funnel asymmetry (*p* = 0.0015).

The motor finding was interpreted as a small-study-effect signal rather than definitive evidence of publication bias because funnel asymmetry can also arise from marked heterogeneity and other study characteristics. The absence of a formal EF test was likewise not interpreted as evidence that publication bias was absent.

### 3.8. Dose–Response and Meta-Regression Analyses

**Figure 5:**
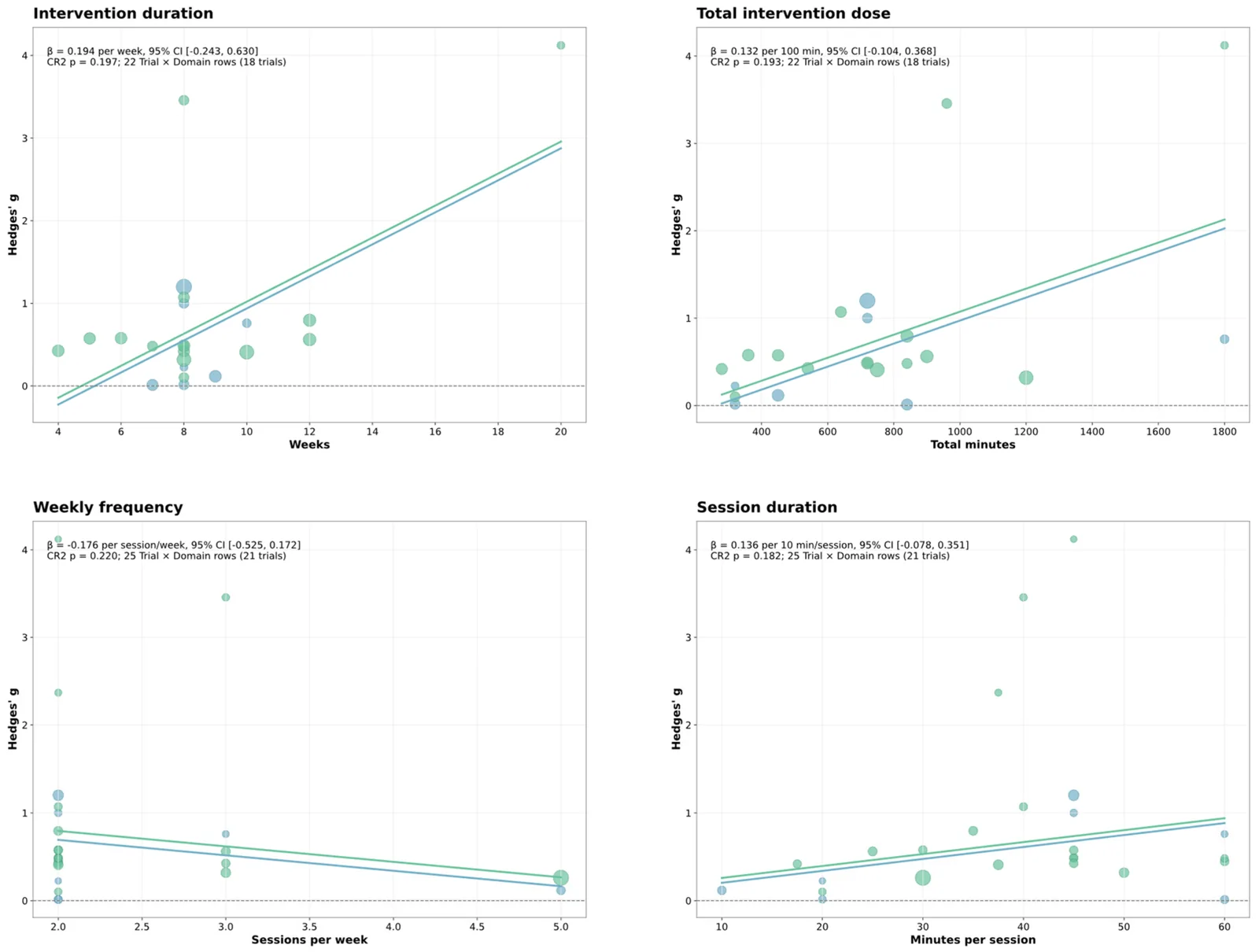
Exploratory study-level dose–response meta-regressions after dependency-aware Trial × Domain aggregation. Blue circles represent executive-function trial-domain estimates and green circles represent motor-competence trial-domain estimates; fitted lines show the corresponding domain-adjusted linear trends. Reported *p* values use trial-clustered CR2 inference. No nonlinear spline models were retained in the analysis.

After dependency-aware aggregation, intervention duration and total intervention minutes were each analyzed using 22 Trial × Domain rows from 18 unique trials, while weekly frequency and session duration were analyzed using 25 rows from 21 trials. In outcome-domain-adjusted CR2 models, none of the four dose variables showed a statistically supported association with effect magnitude: intervention duration beta = 0.194 per week (95% CI [−0.243, 0.630], *p* = 0.197), total dose beta = 0.132 per 100 min (95% CI [−0.104, 0.368], *p* = 0.193), weekly frequency beta = −0.176 per session/week (95% CI [−0.525, 0.172], *p* = 0.220), and session duration beta = 0.136 per 10 min (95% CI [−0.078, 0.351], *p* = 0.182; Figure 5). Holm adjustment across the four exploratory tests did not alter this interpretation (adjusted *p* = 0.729 for each test).

Comparator-adjusted sensitivity models likewise showed no statistically supported dose association. Influence analysis indicated that the duration slope was strongly affected by the 20-week Venetsanou trial (Venetsanou et al., 2014): after excluding this trial, the slope decreased from beta = 0.194 to beta = 0.022 per week (*p* = 0.561). These analyses therefore provide, at most, hypothesis-generating study-level trends and do not support causal recommendations to increase intervention duration or accumulated minutes.

### 3.9. GRADE Assessment

Certainty of evidence was reassessed using the result-level RoB 2 judgements and robust domain-specific estimates (Table 2) (Anjos & Ferraro, 2018; Bentley et al., 2023; Chatzihidiroglou et al., 2018; Chatzipanteli et al., 2007; Chatzopoulos et al., 2022; Dalala et al., 2024; Derri et al., 2001; Đošić et al., 2025; Gencigör & Akın, 2024; Hu et al., 2020; Intawachirarat et al., 2026; Jouira et al., 2024; Kapodistria et al., 2021; W. Liu et al., 2025; Oppici et al., 2020; Tiktampanidi et al., 2021; van den Berg et al., 2019; Venetsanou et al., 2014; Williams et al., 2026; Zachopoulou et al., 2004; Zhao et al., 2024; Zinelabidine et al., 2022). Certainty for executive function was rated very low because of serious risk of bias, serious inconsistency, and serious imprecision. Certainty for motor competence was rated low because of serious risk of bias and serious inconsistency; imprecision was not downgraded because 17 independent trials contributed and the CR2-robust confidence interval around the pooled mean excluded the null. The wide prediction interval was considered under inconsistency because it primarily reflects between-trial variation in true effects across settings; the same feature was therefore not used again to downgrade imprecision. Trial-level funnel asymmetry for motor competence was documented as a small-study-effect concern but was not treated alone as definitive publication bias.

## 4. Discussion

### 4.1. Summary of Main Findings

This systematic review used a dependency-aware three-level framework to evaluate dance and rhythmic physical activity in children, with executive function and motor competence treated as separate primary outcomes. The motor-competence synthesis showed a positive average effect (g = 0.95), whereas the executive-function estimate was positive but statistically uncertain under trial-clustered CR2 inference (g = 0.50, 95% CI crossing zero). Prediction intervals crossed zero in both domains, indicating substantial variability across settings. The dose meta-regressions did not provide statistically supported evidence that longer interventions, greater total minutes, higher weekly frequency, or longer sessions produced larger effects.

### 4.2. Outcome-Specific Effects and Mechanisms

The stronger statistical support for motor competence is consistent with the direct motor demands of dance and rhythmic activity. These interventions require spatial displacement, dynamic control of the center of gravity, multi-limb coordination, and synchronization to external rhythmic cues (Hackney et al., 2024; Hänggi et al., 2010; Karpati et al., 2015). Repeated exposure to these demands can provide task-specific opportunities for motor learning and adaptation (Krakauer et al., 2019; Sigmundsson et al., 2017; Wulf & Lewthwaite, 2016). In the present synthesis, however, the wide prediction interval and high between-trial heterogeneity indicate that the magnitude of motor benefits varied substantially, and the low GRADE certainty precludes assuming a uniform effect across programs or settings.

Executive-function effects are more plausibly interpreted as transfer from cognitively demanding movement rather than as the direct target of most interventions (Moreau & Conway, 2014; Noack et al., 2014; Sala & Gobet, 2019). Learning dance sequences requires updating movement representations in working memory, inhibiting competing responses, and switching actions when rhythmic or choreographic demands change (Diamond, 2013; Mao et al., 2024; Richter et al., 2025). These mechanisms remain plausible, but the empirical evidence in this review was limited to seven independent trials. The robust confidence interval crossed zero, influence analyses showed sensitivity of magnitude, and GRADE certainty was very low. Accordingly, the current evidence suggests a possible cognitive benefit but does not establish a reliable executive-function effect.

### 4.3. Moderating Effects of Demographics and Interventions

Categorical moderator analyses were interpreted cautiously because only seven EF trials and 17 MC trials were available, and several prespecified age, intervention-type, and comparator categories contained too few independent trials for stable multivariable inference. Differences were therefore not interpreted simply because one subgroup estimate was statistically significant and another was not.

This restraint does not imply that intervention form or participant characteristics are irrelevant. Rather, the current evidence base is too sparse to distinguish reliably among specific dance genres, age strata, or comparator conditions after accounting for trial-level dependence. Future trials using consistent outcome measures and clearer reporting of intervention content will be better suited to testing whether rhythmic cueing, sequencing complexity, or particular dance formats modify effects.

### 4.4. Dose–Response Relationship

In the dependency-aware dose–response analyses, after outcomes were aggregated so that trials reporting many measures did not receive disproportionate exposure weight, and after CR2 small-sample inference was applied, neither intervention duration nor total minutes was statistically associated with effect magnitude. Their positive point estimates were accompanied by wide confidence intervals, and the duration slope was strongly influenced by one long-duration trial. Weekly frequency and session duration were likewise not associated with effect size.

These findings are compatible with the possibility that repeated practice contributes to motor learning and consolidation (Dayan & Cohen, 2011; Doyon et al., 2009; Hillman et al., 2008; Kantak & Winstein, 2012; Lubans et al., 2016; Robertson et al., 2004; Schmidt, 1975; Valkenborghs et al., 2019), but the present study-level meta-regressions cannot establish that increasing dose causes larger benefits. Intervention duration, total minutes, participant characteristics, intervention content, and study design may be correlated across trials. Dose findings should therefore be regarded as exploratory and hypothesis-generating rather than as a basis for prescriptive thresholds.

### 4.5. Limitations

Several limitations should be considered. First, heterogeneity remained very high after accounting for dependence and cluster design, and prediction intervals crossed zero in both domains. Differences in intervention content, comparator conditions, outcome instruments, and participant characteristics may contribute to this variability. Although the included interventions shared structured rhythmic cueing, repeated motor sequencing, and coordination demands, they encompassed heterogeneous formats, including creative and aerobic dance, action–song, digital dance, and music-and-movement programs. They should therefore not be interpreted as a homogeneous intervention class, and the positive average motor-competence effect should be understood as an average across diverse programs rather than a uniform effect attributable to a single intervention type. Second, result-level RoB 2 assessments identified methodological concerns across most trials, including two high-risk MC trials, and GRADE certainty was very low for EF and low for MC. Third, EF evidence was based on only seven independent trials, limiting precision and precluding extensive moderator or small-study-effect testing. Fourth, some effect-size calculations required reconstructed change-score SDs, assumed ICCs, or study-specific conversions; although sensitivity analyses did not change the domain-level inference, the magnitude of pooled effects varied under alternative assumptions. Finally, motor-competence funnel asymmetry suggests possible small-study effects, but this signal is not specific to publication bias in the presence of substantial heterogeneity.

### 4.6. Practical Applications

From a practical perspective, dance and rhythmic physical activity may be considered as potentially useful movement-based options for supporting motor competence in school and community settings. However, this interpretation should remain cautious because certainty of evidence is low, heterogeneity is substantial, the prediction interval crosses the null, and a small-study-effect signal was detected. The current evidence does not identify a single superior dance format or dose. Program choice should therefore be guided by feasibility, age-appropriate movement demands, available instruction, and the extent to which the activity provides structured rhythmic cueing, sequencing, and coordination practice.

For executive function, practical claims should be more limited because the current estimate remains uncertain and the certainty of evidence is very low. Likewise, the study-level dose analyses do not justify prescribing longer interventions or higher accumulated minutes as inherently more effective. Future trials should directly compare dose levels within the same design and should prespecify both motor and core executive-function outcomes before stronger implementation recommendations are made.

## 5. Conclusions

This systematic review and three-level meta-analysis indicates that dance and rhythmic physical activity may improve motor competence in children on average, although the magnitude of benefit varies substantially across settings and the certainty of evidence is low. For core executive function, the average estimate was positive but statistically uncertain and supported by very-low-certainty evidence. The dose analyses did not establish that longer interventions or greater total doses produce larger effects. More rigorous randomized trials with transparent allocation, appropriate handling of clustered designs, consistent core outcomes, and prespecified analyses are needed to strengthen conclusions about both motor and cognitive effects.

## Figures and Tables

**Table 1:** Basic characteristics of the included reports and underlying trials.

Report	Country	Population	N	Age/School Level	Intervention	Comparator	Dose	Outcomes
Anjos and Ferraro (2018)	Brazil	Healthy first-year elementary-school children	N = 85; IG = 51, CG = 34	~6.5 years; Grade 1	Educational dance/Laban-based creative dance	Usual curriculum	7 months; 2×/week; 60 min	MC: motor development, balance, fine/gross motor skills
Bentley et al. (2023)	Australia	Preschool children from disadvantaged communities	N = 213; IG = 112, CG = 101	Mean 50.54 months; preschool	RAMSR rhythm and movement program	Usual preschool program	8 weeks; 16–20 sessions; ~20 min	EF: inhibition, working memory, shifting; MC: visual-motor integration
van den Berg et al. (2019)	Netherlands	Primary-school children, grades 5–6	N = 512; IG = 263, CG = 249	9–12 years	Just Dance classroom exercise breaks	Educational active control	9 weeks; ~5×/week; 10 min	EF: attention, inhibition, executive control
Chatzihidiroglou et al. (2018)	Greece	Healthy preschool children	N = 42; IG = 22, CG = 20	54–78 months; preschool	Creative dance program	Free-play motor activities	8 weeks; 2×/week; 45 min	MC: rhythm, balance, reaction time
Chatzipanteli et al. (2007)	Greece	First-grade primary-school children	N = 75; IG = 40, CG = 35	Mean 6.3 years; Grade 1	Music-movement program	Same movement program without music	8 weeks; 2×/week; 35–45 min	MC: TGMD-2 object-control skills
Chatzopoulos et al. (2022)	Greece	Healthy preschool children	N = 51; IG = 25, CG = 26	Mean ~4.9 years; preschool	Action–song balance training	Regular PE	6 weeks; 2×/week; 30 min	MC: static/dynamic balance, strength
Dalala et al. (2024)	Greece	Nursery-school children	N = 39; IG = 18, CG = 21	Mean 3.35 years; preschool	Interactive/action–song balance program	Standard PE	8 weeks; 2×/week; 15–20 min	MC: static balance, dynamic balance
Derri et al. (2001)	Greece	Preschool children	N = 68; IG = 35, CG = 33	4–6 years; preschool	Music and movement program	Free-play movement activities	10 weeks; 2×/week; 35–40 min	MC: TGMD locomotor skills
Đošić et al. (2025)	Serbia	Children from preschool institutions	N = 80; EG1 = 26, EG2 = 26, CG = 28	Mean ~6.5 years	Structured dance program; two dose arms	Standard PE	12 weeks; EG1: 2 × 35 min/week; EG2: 3 × 25 min/week	MC: BOT-2 motor competence domains
Gencigör and Akın (2024)	Türkiye	First-grade students	N = 78; IG = 39, CG = 39	~7 years; Grade 1	Orff-based movement education	Usual curriculum	8 weeks; 3×/week; 40 min	MC: TGMD-2 locomotor and object-control skills
Hu et al. (2020)	China	Kindergarten children	N = 289; IG = 142, CG = 147	3–5 years; preschool	Novel rhythmic physical activities	Traditional rhythmic physical activities	One school year; 5×/week; 30 min	MC: gross motor skills, static/dynamic balance
Intawachirarat et al. (2026)	Thailand	Children with obesity, grades 3–6	N = 35; IG = 17, CG = 18	9–12 years	Contemporary dance training	Active school-activity control	10 weeks; 3×/week; 60 min	EF: working memory, inhibition, flexibility, planning
Jouira et al. (2024) †	Tunisia	Primary-school children with low PA	N = 41; IG = 19, CG = 22	9–11 years	Aerobic dance training	No PE/usual activities	8 weeks; 2×/week; 45 min	MC: postural balance
Kapodistria et al. (2021)	Greece	Primary-school children	N = 61; IG = 31, CG = 30	Mean 6.41 years	Greek traditional dance program	Regular PE	4 weeks; 3×/week; 45 min	MC: sensorimotor synchronization, reaction time
W. Liu et al. (2025)	China	Healthy kindergarten children	N = 201; IG = 97, CG = 104	4–6 years; preschool	Early childhood DanceSport	Usual kindergarten activities	8 weeks; 2×/week; 45 min	EF: cognitive flexibility, inhibition, working memory
Oppici et al. (2020)	Australia	Primary-school children, grades 3–4	N = 80; high-cognitive = 30, low-cognitive = 30, CG = 20	8–10 years	High- and low-cognitive jazz-dance programs	Standard PE/sport curriculum	7 weeks; 2×/week; ~60 min	EF: working memory, flexibility, inhibition; MC: CAMSA
Tiktampanidi et al. (2021)	Greece	Preschool children	N = 50; IG = 25, CG = 25	~52–54 months; preschool	Laban-based creative movement program	Regular PE	5 weeks; 2×/week; 45 min	MC: static balance, dynamic balance, TUG
Venetsanou et al. (2014)	Greece	Preschool children	N = 70; IG = 36, CG = 34	48–72 months	Music and movement program	No organized PA	20 weeks; 2×/week; 45 min	MC: motor rhythmic ability
Williams et al. (2026)	China, Hong Kong SAR	Low-SES K2 kindergarten children	N = 286; IG = 129, CG = 157	46–62 months; K2	RAMSR Hong Kong adaptation	Active control materials	8 weeks; 2×/week; ~20 min	EF: working memory, inhibition, shifting, self-regulation
Zachopoulou et al. (2004)	Greece	Preschool children	N = 90; IG = 50, CG = 40	4–6 years	Orff-based music and movement program	PE without rhythmic accompaniment	2 months; 2×/week; 35–40 min	MC: jumping, dynamic balance
Zhao et al. (2024)	China	Kindergarten children	N = 50; IG = 25, CG = 25	4–5 years	Rhythmic physical activity	Usual lifestyle/no intervention	8 weeks; 3×/week; 50 min	MC: gross motor skills, balance, locomotor and object-control skills
Zinelabidine et al. (2022) †	Tunisia	Primary-school children with low PA	N = 41; IG = 19, CG = 22	9–11 years	Aerobic dance program	No PE/normal daily activities	8 weeks; 2×/week; 45 min	EF: cognitive flexibility, inhibition, working memory

Note. IG = intervention group; CG = control group; EG = experimental group; EF = executive function; MC = motor competence; PA = physical activity; PE = physical education. † Jouira et al. (2024) and Zinelabidine et al. (2022) are separate reports from the same underlying randomized trial; their 41 participants were counted once in the overall unique sample.

**Table 2:** Summary of findings and certainty of evidence.

Outcome	Studies/Effect Sizes	Participants	Risk of Bias	Inconsistency	Indirectness	Imprecision	Publication Bias	Certainty of Evidence
Executive function	7/34	1368	Serious ^a^	Serious ^b^	Not serious ^c^	Serious ^d^	Not downgraded ^e^	Very low
Motor competence	17/103	1462	Serious ^f^	Serious ^g^	Not serious ^c^	Not serious ^h^	Not downgraded ^i^	Low

Note: ^a^ Serious risk of bias: all seven EF trial-level result assessments had some concerns. ^b^ Serious inconsistency: the EF prediction interval was wide and crossed the null (−1.24 to 2.24). ^c^ Not serious indirectness: the review question and included populations/outcomes were aligned. ^d^ Serious imprecision: only seven EF trials contributed, and the CR2-robust CI crossed zero. ^e^ Publication bias not downgraded: no formal asymmetry test was considered reliable with seven trials. ^f^ Serious risk of bias for MC: 1/17 trial-level assessments was low risk, 14/17 had some concerns, and 2/17 were high risk. ^g^ Serious inconsistency: the MC prediction interval was wide and crossed the null (−1.43 to 3.34). ^h^ Imprecision not serious: 17 independent trials contributed, and the CR2-robust CI around the pooled mean excluded zero. The wide prediction interval was considered under inconsistency because it reflects between-trial variation in true effects and was not used again to downgrade imprecision. ^i^ Publication bias not downgraded, with concern noted: trial-level asymmetry was detected (*p* = 0.0015) but was not regarded as specific evidence of publication bias in the presence of marked heterogeneity.

## Data Availability

The data supporting the findings of this study are available within the article and its Supplementary Materials. Further inquiries can be directed to the corresponding author.

## Supplementary Materials

The following supporting information can be downloaded at: https://www.mdpi.com/article/10.3390/bs16091699/s1: Supplemental S1: Search Strategy; Supplemental S2: Supplementary Figures; Supplemental S3: Supplementary Tables.
