# Supplementary material for: Effects of Dance and Rhythmic Physical Activity Interventions on Executive Function and Motor Competence in Children: A Systematic Review and Three-Level Meta-Analysis
Source: Behav Sci (Basel). 2026 Sep 20;16(9):1699. doi: 10.3390/bs16091699 (PMC13603710; doi:10.3390/bs16091699)
Supplement: Supplementary file 1 [file behavsci-16-01699-s001.zip › Supplement Materials.pdf]

Effects of Dance and Rhythmic Physical Activity Interventions on Executive Function and Motor Competence in Children: A Systematic Review and Three-Level Meta-Analysis

Supplemental S1: Search Strategy (Pages 1-5)

Supplemental S2: Supplementary Figures (Pages 6-12)

Supplemental S3: Supplementary Tables (Pages 13-21)

Supplemental S1: Search Strategy

1. PubMed final search strategy, 2026-05-26, n=352

(("Dancing"[Mesh] OR dance[tiab] OR dances[tiab] OR dancing[tiab] OR dance-based[tiab] OR "creative dance"[tiab] OR "educational dance"[tiab] OR "folk dance"[tiab] OR "traditional dance"[tiab] OR "aerobic dance"[tiab] OR "sport dance"[tiab] OR dancesport[tiab] OR "dance sport"[tiab] OR "digital dance"[tiab] OR "dance game"[tiab] OR "dance games"[tiab] OR "Just Dance"[tiab] OR exergam*[tiab] OR "rhythmic physical activity"[tiab] OR "rhythmic physical activities"[tiab] OR "rhythm and movement"[tiab] OR "music and movement"[tiab] OR "movement education"[tiab] OR "Orff"[tiab] OR "Laban"[tiab] OR "action song"[tiab] OR "action songs"[tiab]) AND ("Child"[Mesh] OR "Adolescent"[Mesh] OR child[tiab] OR children[tiab] OR childhood[tiab] OR preschool*[tiab] OR kindergarten[tiab] OR schoolchild*[tiab] OR "school-age"[tiab] OR student*[tiab] OR adolescent[tiab] OR adolescents[tiab] OR youth[tiab] OR youths[tiab] OR teen*[tiab] OR pediatric[tiab] OR paediatric[tiab]) AND ("Executive Function"[Mesh] OR "Cognition"[Mesh] OR "Cognitive Dysfunction"[Mesh] OR "Motor Skills"[Mesh] OR "Psychomotor Performance"[Mesh] OR "Postural Balance"[Mesh] OR "Physical Functional Performance"[Mesh] OR "executive function"[tiab] OR "executive functions"[tiab] OR inhibition[tiab] OR inhibitory[tiab] OR "inhibitory control"[tiab] OR "working memory"[tiab] OR "cognitive flexibility"[tiab] OR shifting[tiab] OR attention[tiab] OR "self-regulation"[tiab] OR "motor competence"[tiab] OR "motor competency"[tiab] OR "motor skill"[tiab] OR "motor skills"[tiab] OR "motor development"[tiab] OR "gross motor"[tiab] OR "fine motor"[tiab] OR locomotor[tiab] OR "object control"[tiab] OR balance[tiab] OR coordination[tiab] OR "visual-motor"[tiab] OR "visual motor"[tiab] OR TGMD[tiab] OR "BOT-2"[tiab] OR "Movement ABC"[tiab] OR MABC[tiab])) AND (randomized controlled trial[pt] OR controlled clinical trial[pt] OR random*[tiab] OR trial[tiab] OR intervention[tiab] OR program[tiab] OR programme[tiab])

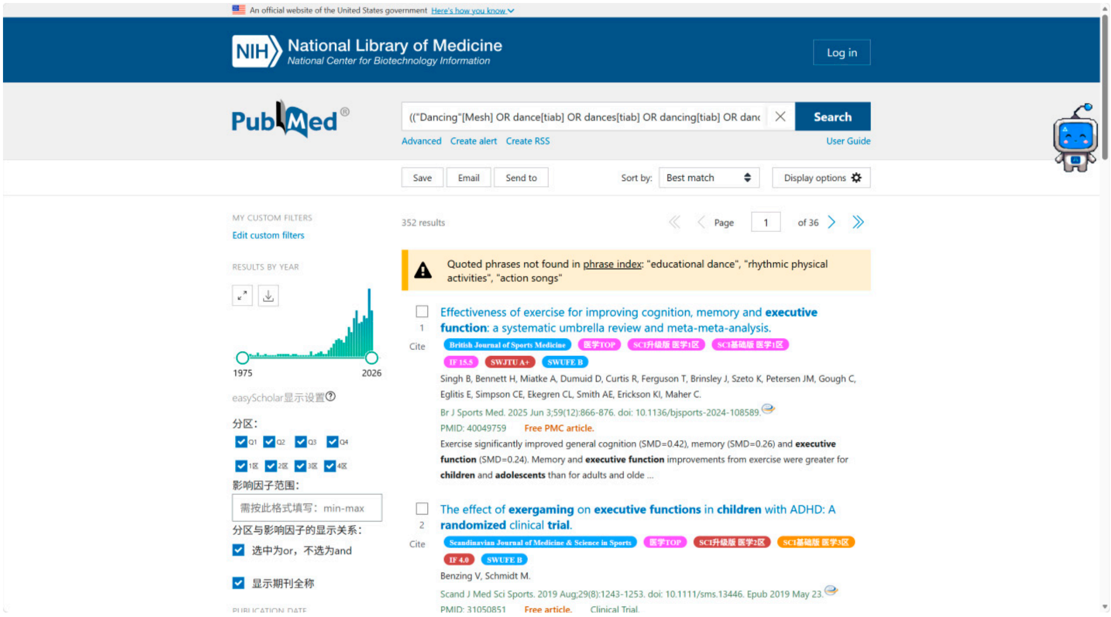

2.Web of Science Core Collection, 2026-05-26, n=775

TS=(dance OR dances OR dancing OR "dance-based" OR "creative dance" OR "educational dance" OR "folk dance" OR "traditional dance" OR "aerobic dance" OR "sport dance" OR dancesport OR "dance sport*" OR "digital dance" OR "dance game*" OR "Just Dance" OR exergam* OR "rhythmic physical activit*" OR "rhythm and movement" OR "music and movement" OR "movement education" OR "action song*" OR (Orff NEAR/3 (movement OR music OR dance)) OR (Laban NEAR/3 (movement OR dance))) AND TS=(child* OR preschool* OR kindergarten* OR schoolchild* OR "school-age" OR "school aged" OR student* OR adolescent* OR youth* OR teen* OR pediatric OR paediatric) AND TS=("executive function*" OR cognition OR cognitive OR inhibition OR inhibitory OR "inhibitory control" OR "working memory" OR "cognitive flexibility" OR shifting OR attention OR "self-regulation" OR "motor competence" OR "motor competency" OR "motor skill*" OR "motor development" OR "gross motor" OR "fine motor" OR locomotor OR "object control" OR balance OR coordination OR "visual-motor" OR "visual motor" OR TGMD OR "BOT-2" OR "Movement ABC" OR MABC) AND TS=(random* OR trial OR intervention OR program OR programme OR training OR experiment*)

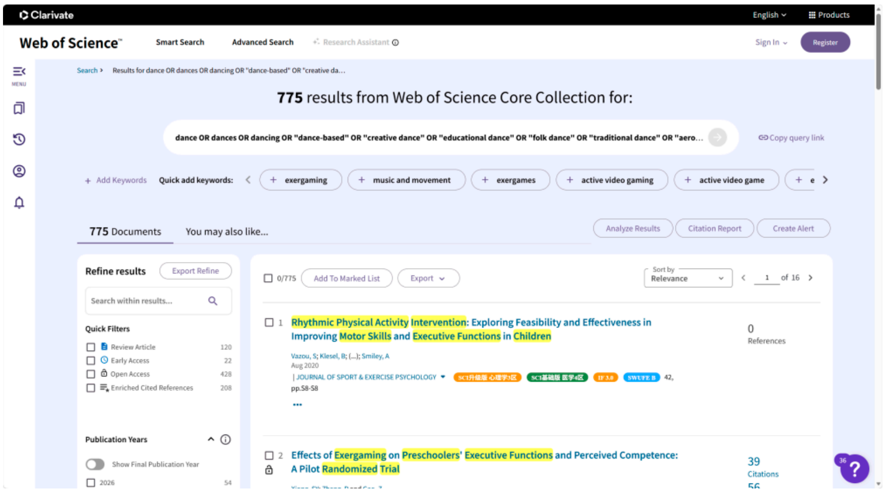

3.Scopus, 2026-05-26, n=1587

TITLE-ABS-KEY((dance OR dances OR dancing OR "dance-based" OR "creative dance" OR "educational dance" OR "folk dance" OR "traditional dance" OR "aerobic dance" OR "sport dance" OR dancesport OR "dance sport*" OR "digital dance" OR "dance game*" OR "Just Dance" OR exergam* OR "rhythmic physical activit*" OR "rhythm and movement" OR "music and movement" OR "movement education" OR "action song*" OR Orff OR Laban) AND (child* OR preschool* OR kindergarten* OR schoolchild* OR "school-age" OR "school aged" OR student* OR adolescent* OR youth* OR teen* OR pediatric OR paediatric) AND ("executive function*" OR cognition OR cognitive OR inhibition OR inhibitory OR "inhibitory control" OR "working memory" OR "cognitive flexibility" OR shifting OR attention OR "self-regulation" OR "motor competence" OR "motor competency" OR "motor skill*" OR "motor development" OR "gross motor" OR "fine motor" OR locomotor OR "object control" OR balance OR coordination OR "visual-motor" OR "visual motor" OR TGMD OR "BOT-2" OR "Movement ABC" OR MABC) AND (random* OR trial OR intervention OR program OR programme OR training OR experiment*))

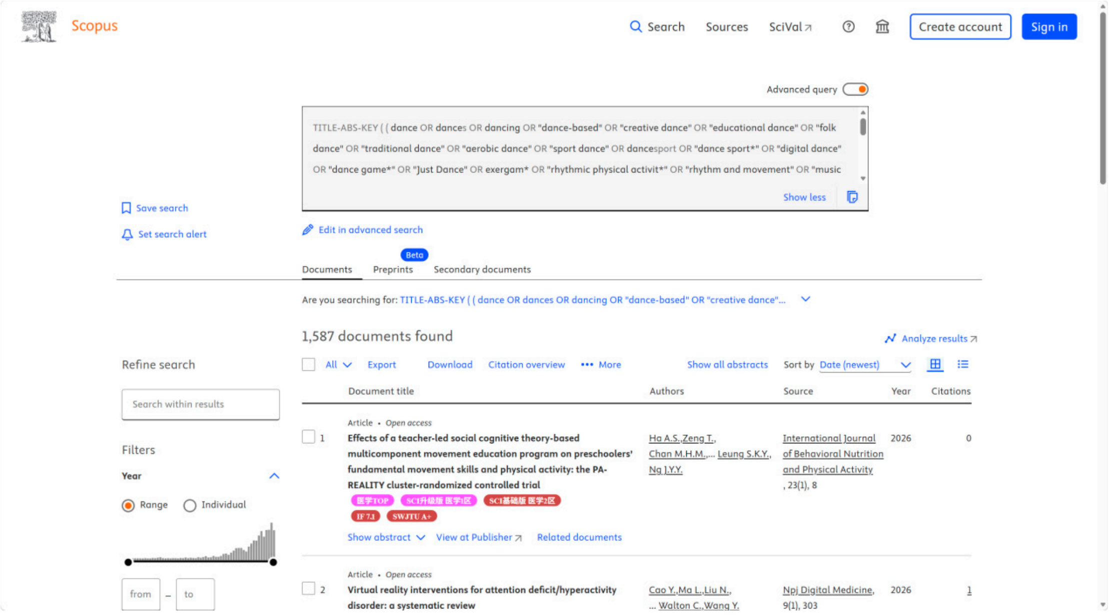

4.ProQuest Dissertations and Theses Global, 2026-05-27, n=502

abstract("dance" OR "dances" OR "dancing" OR "dance-based" OR "creative dance" OR "educational dance" OR "folk dance" OR "traditional dance" OR "aerobic dance" OR "sport dance" OR dancesport OR "dance sport" OR "digital dance" OR "dance game" OR "dance games" OR "Just Dance" OR exergam* OR "rhythmic physical activity" OR "rhythmic physical activities" OR "rhythm and movement" OR "music and movement" OR "movement education" OR "action song" OR "action songs" OR Orff OR Laban) AND abstract(child* OR preschool* OR kindergarten* OR schoolchild* OR "school-age" OR "school aged" OR student* OR adolescent* OR youth* OR teen* OR pediatric OR paediatric) AND abstract("executive function" OR "executive functions" OR cognition OR cognitive OR inhibition OR inhibitory OR "inhibitory control" OR "working memory" OR "cognitive flexibility" OR shifting OR attention OR "self-regulation" OR "motor competence" OR "motor competency" OR "motor skill" OR "motor skills" OR "motor development" OR "gross motor" OR "fine motor" OR locomotor OR "object

control" OR balance OR coordination OR "visual-motor" OR "visual motor" OR TGMD OR "BOT-2" OR "Movement ABC" OR MABC) AND abstract(random* OR trial OR intervention OR program OR programme OR training OR experiment*)

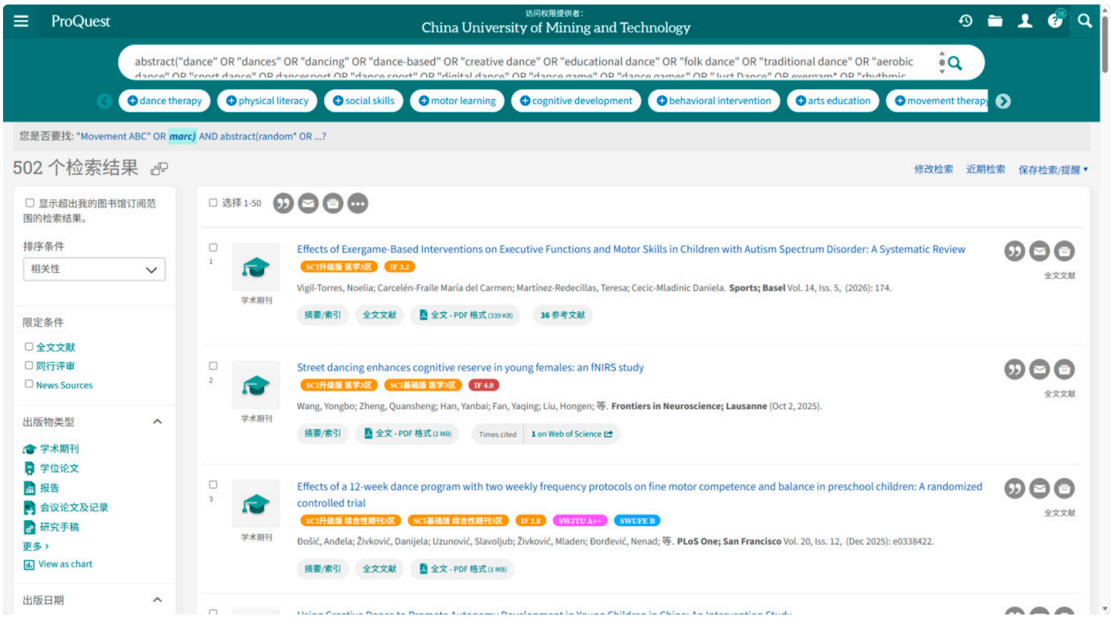

5.ERIC, 2026-05-27, n=576

(dance OR dancing OR "creative dance" OR "educational dance" OR "folk dance" OR "traditional dance" OR "aerobic dance" OR dancesport OR "digital dance" OR "dance game" OR "Just Dance" OR exergame OR exergaming OR "rhythmic physical activity" OR "rhythm and movement" OR "music and movement" OR "movement education" OR "action song" OR Orff OR Laban) AND (child OR children OR preschool OR kindergarten OR schoolchildren OR "school age" OR students OR adolescent OR adolescents OR youth OR pediatric) AND ("executive function" OR cognition OR inhibition OR "inhibitory control" OR "working memory" OR "cognitive flexibility" OR attention OR "self regulation" OR "motor competence" OR "motor skills" OR "motor development" OR "gross motor" OR "fine motor" OR locomotor OR balance OR coordination) AND (randomized OR randomised OR trial OR intervention OR program OR programme OR training OR experiment)

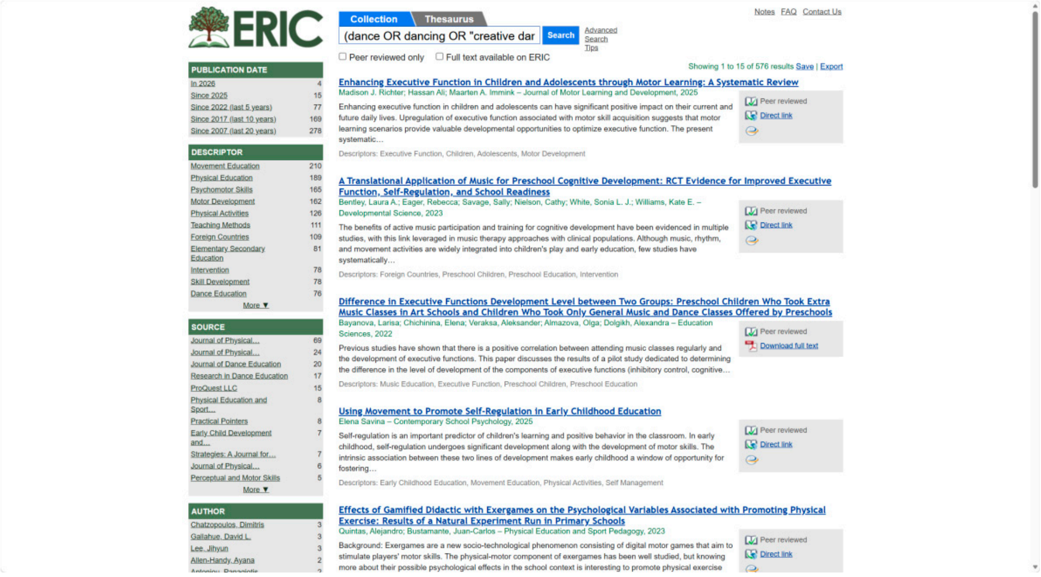

6.ClinicalTrials.gov, 2026-05-27, n=121

(dance OR dancing OR dancesport OR "creative dance" OR "aerobic dance" OR "rhythmic physical activity" OR "rhythm and movement" OR "music and movement" OR "movement education" OR exergame OR exergaming OR "Just Dance") AND (child OR children OR preschool OR kindergarten OR adolescent OR youth) AND ("executive function" OR "working memory" OR inhibition OR "motor skill" OR "motor skills" OR "motor competence" OR balance OR coordination)

Supplemental S2: Supplementary Figures

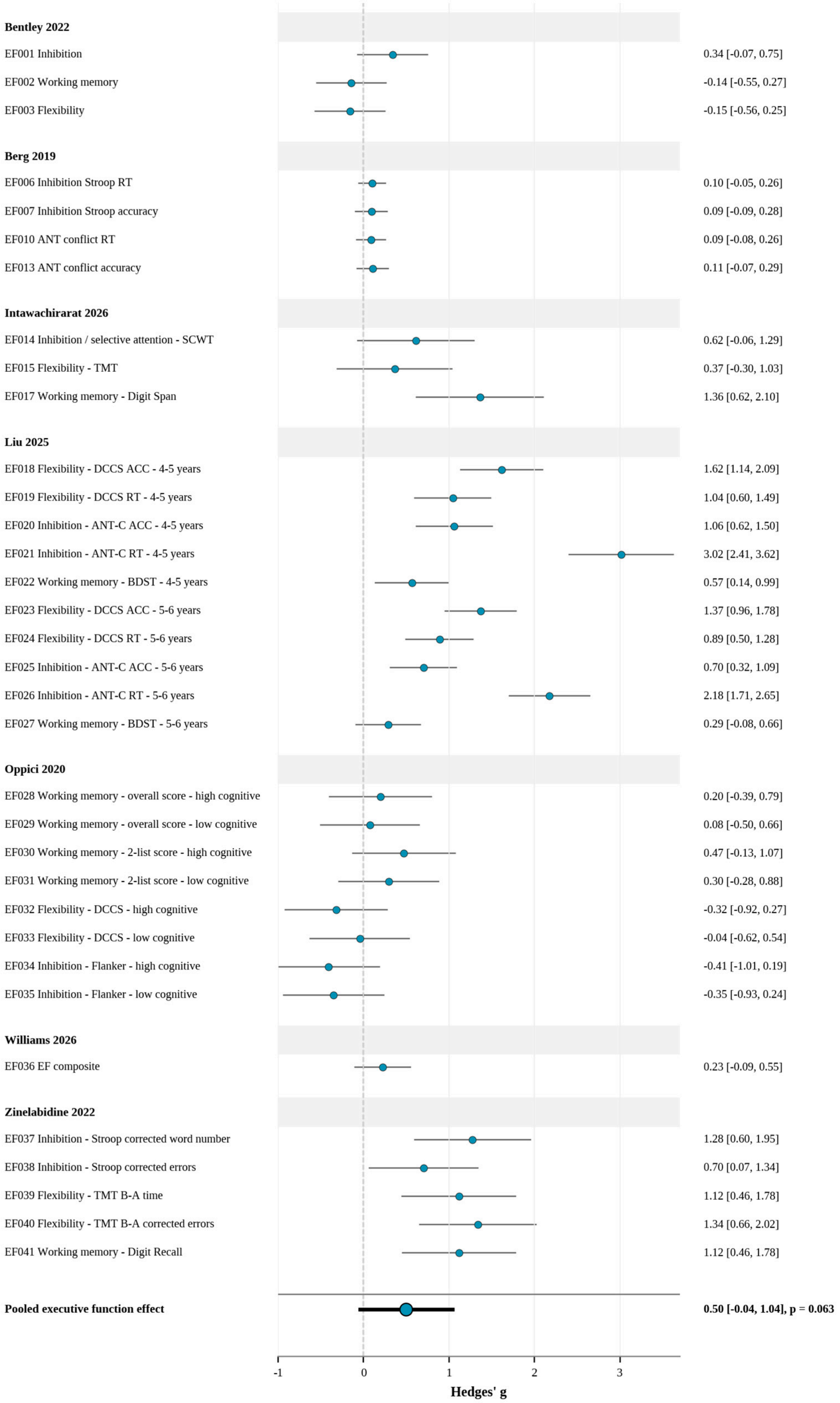

Figure S1. Forest plot of the primary core executive-function effects.

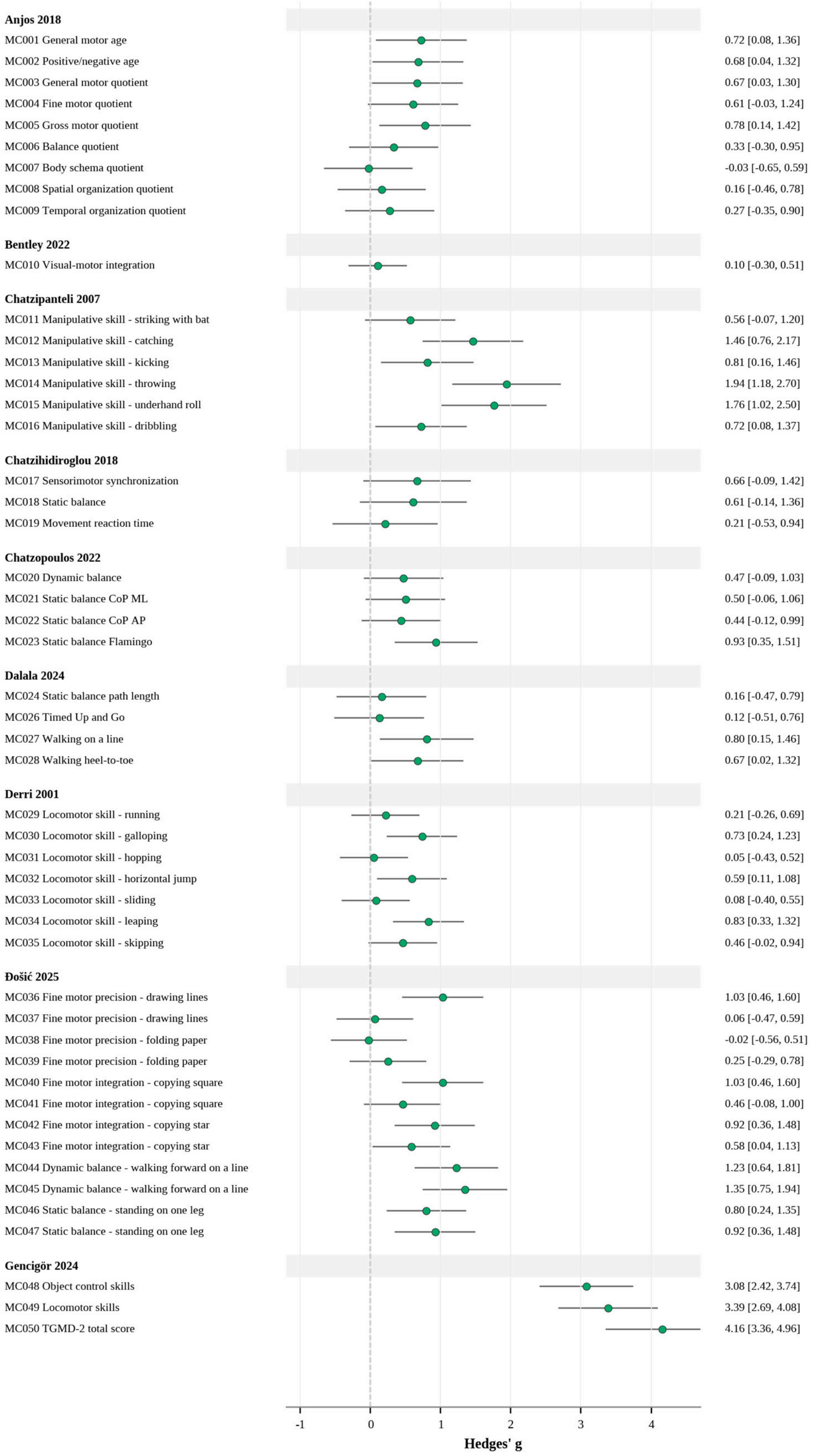

Figure S2A. Forest plot of primary motor-competence effect sizes (part A).

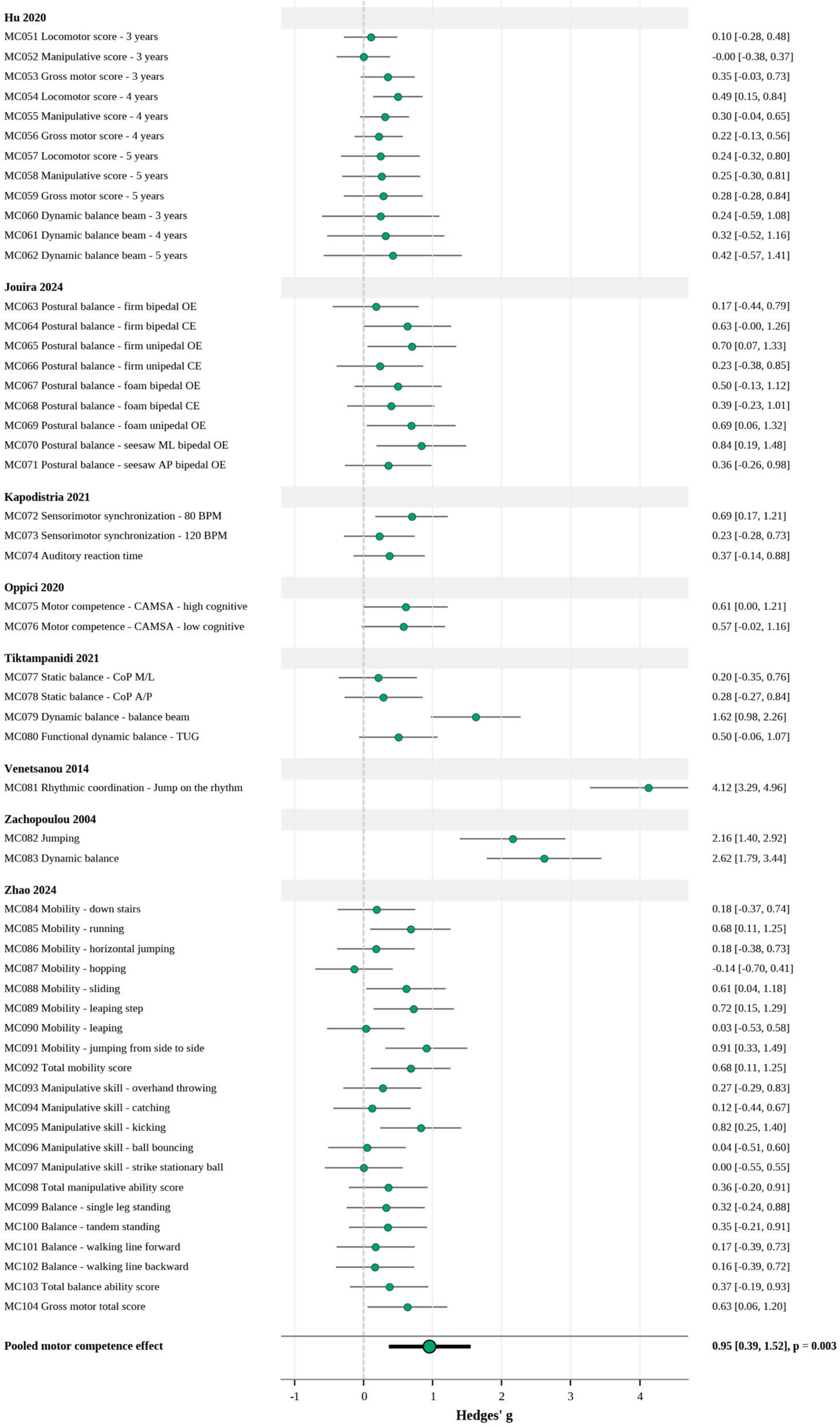

Figure S2B. Forest plot of primary motor-competence effect sizes (part B).

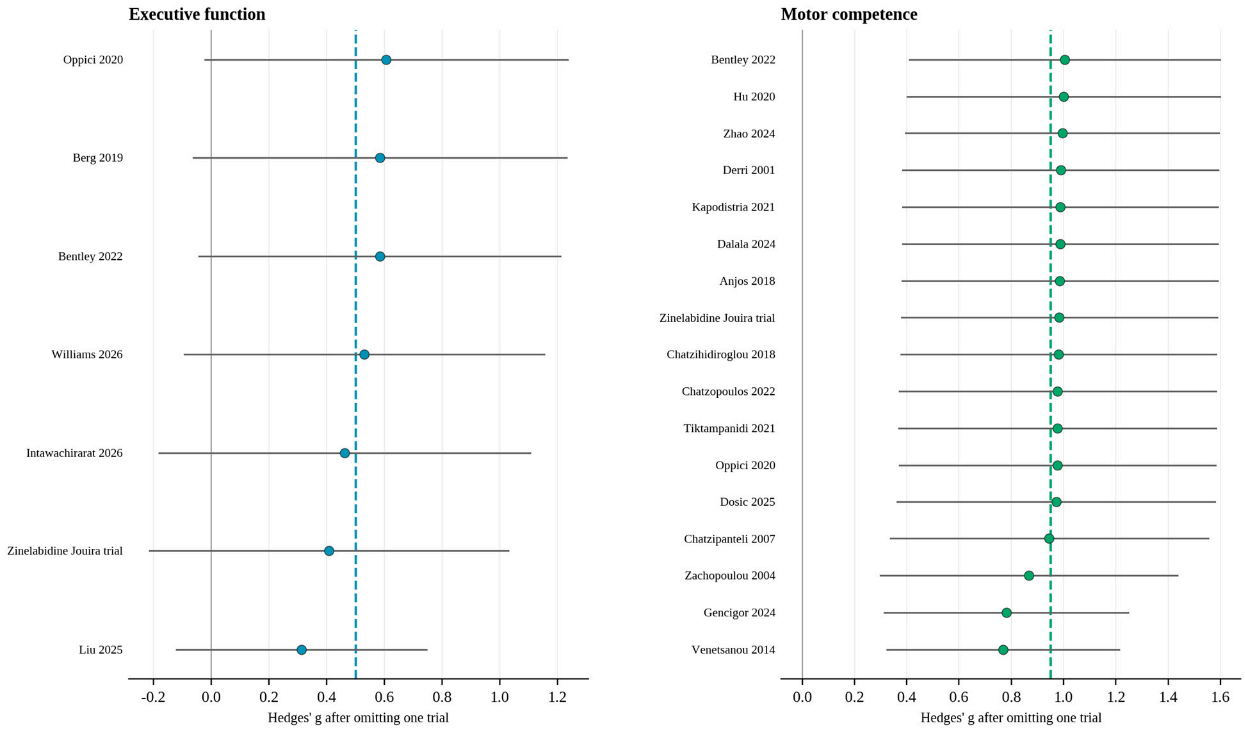

Figure S3. Leave-one-trial-out sensitivity analyses for executive function and motor competence.

Note. Each point is the pooled estimate after omitting one underlying trial. Dashed vertical lines indicate the corresponding full-model estimate. Jouira 2024 and Zinelabidine 2022 were treated as reports from one underlying trial.

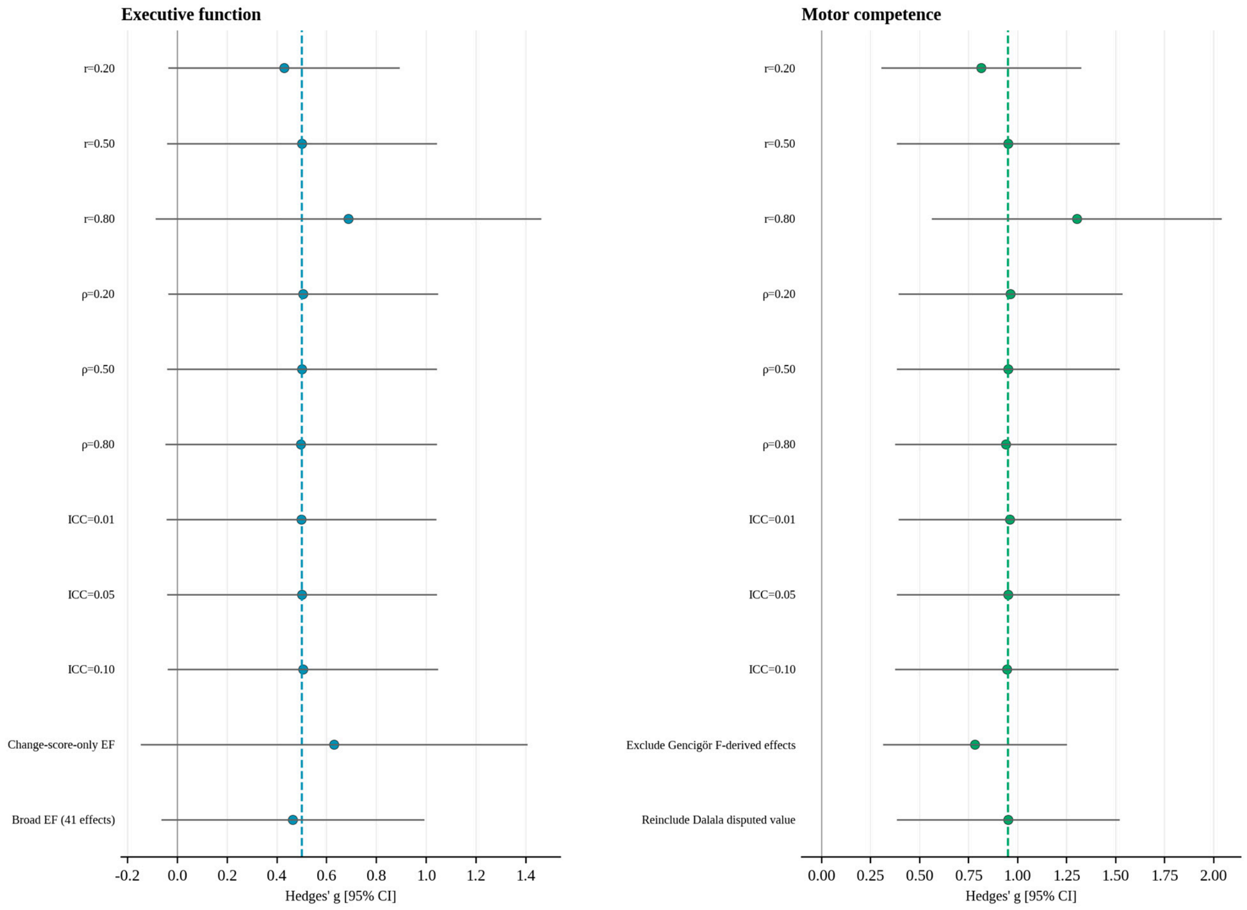

Figure S4. Sensitivity analyses under alternative correlation, clustering, construct, and source-data assumptions.

Note. r denotes the assumed pre-post correlation used to reconstruct change-score SDs; rho denotes the working within-cohort sampling correlation; ICC denotes the assumed intracluster correlation for cluster trials without a reported value. Dashed lines show the primary estimates. Domain-level inference was unchanged across these assumptions.

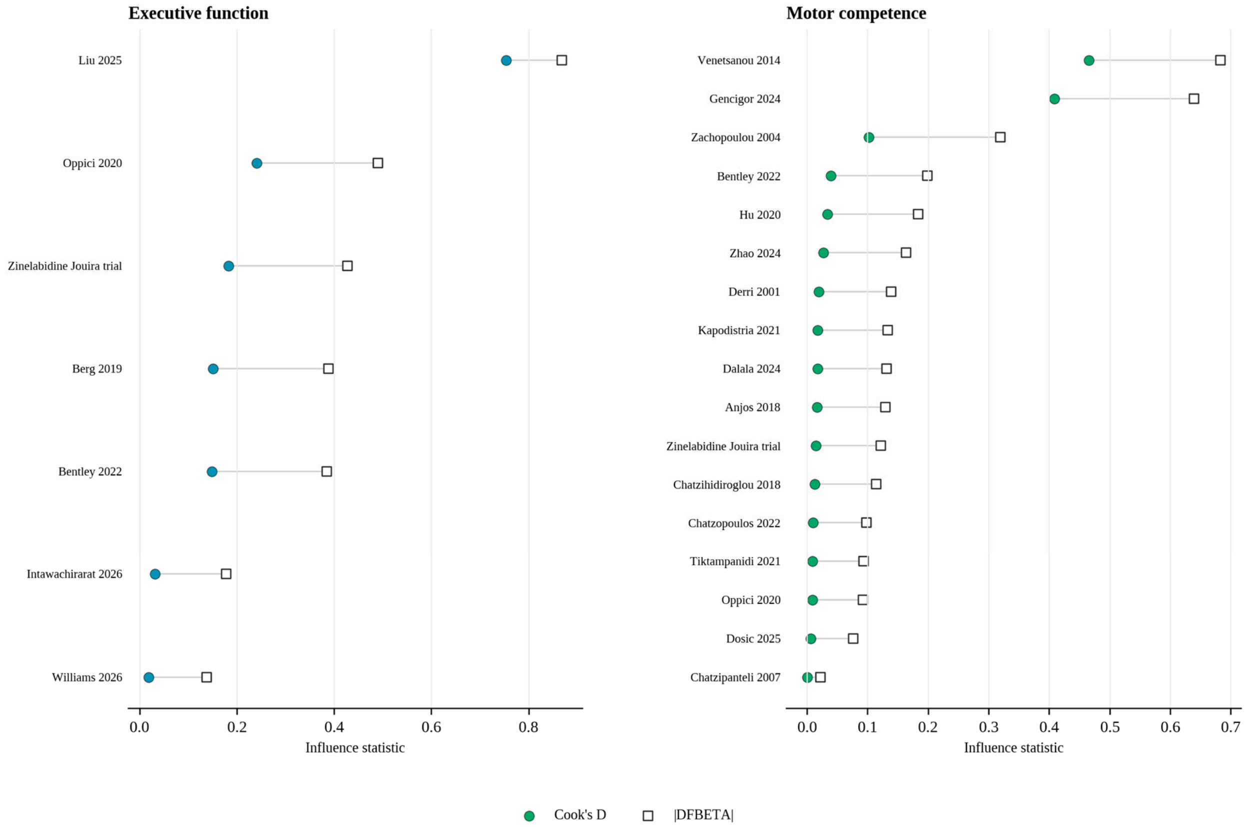

Figure S5. Trial-level influence diagnostics for the two primary outcome domains.

Note. Cook's D and absolute DFBETA are shown for each underlying trial. Liu 2025 was the most influential EF trial; Venetsanou 2014 and Gencigör 2024 had the largest influence statistics in the MC model. Influence diagnostics were used to guide sensitivity analyses rather than to delete effects according to an arbitrary Hedges' g threshold.

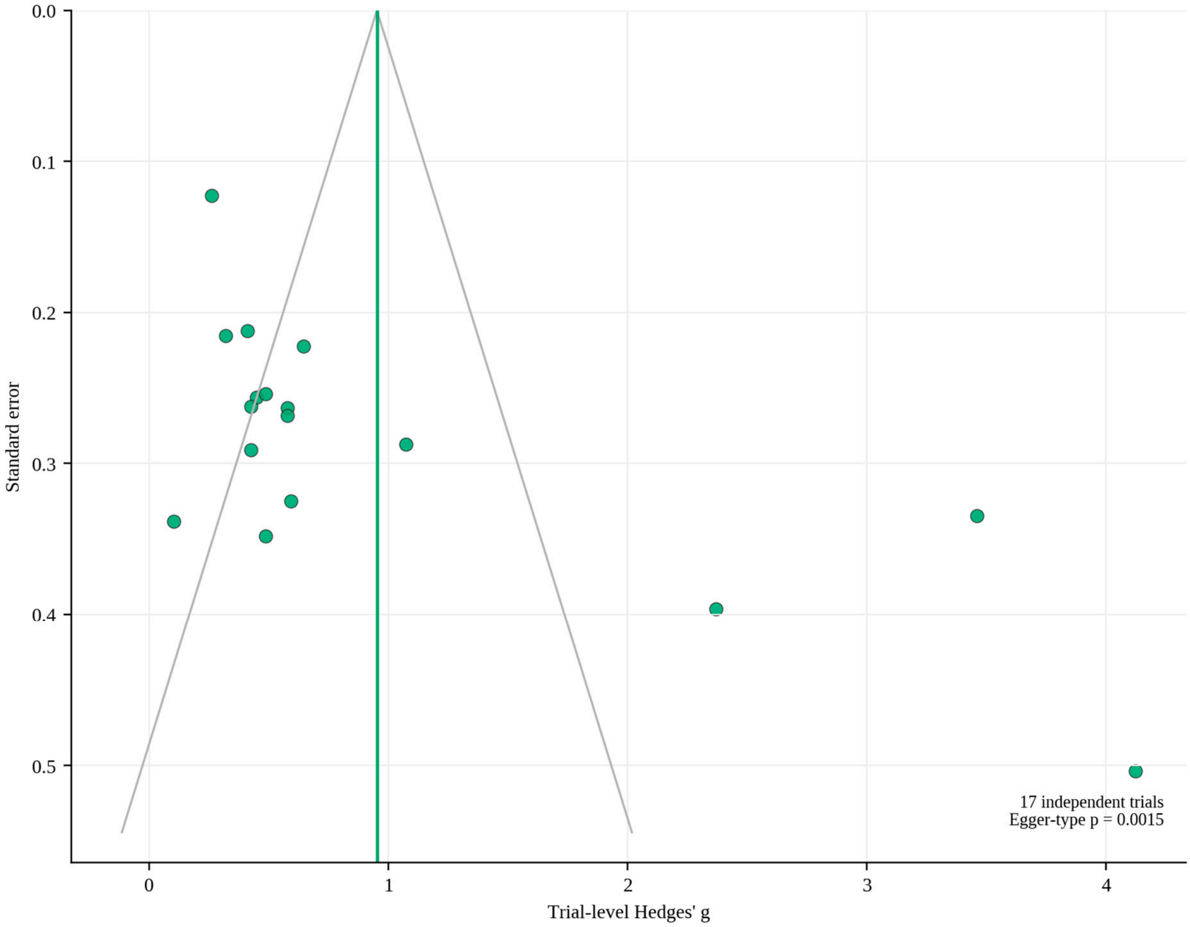

Figure S6. Trial-level funnel plot for motor competence.

Note. The 103 dependent motor effects were aggregated to 17 covariance-aware trial-level estimates before the asymmetry analysis. The Egger-type regression indicated funnel asymmetry (p = 0.0015). This was interpreted as a small-study-effect signal rather than definitive evidence of publication bias because heterogeneity remained substantial. No formal asymmetry test was conducted for executive function because only seven independent trials contributed.

Supplemental S3: Supplementary Tables

Table S1. Trial/report structure and design handling in the revised analysis.

Report	Trial ID	Domain	Design / randomization unit	Dependency issue	Source adjustment	Revised analysis handling
Anjos 2018	Anjos_2018	Motor competence	Cluster RCT / Classroom	Clustered design	No; t-tests/chi-square used	Apply design-effect correction; ICC sensitivity
Bentley 2022	Bentley_2022	Executive function + Motor competence	Cluster RCT / Preschool center	Clustered design	Yes; multilevel model with child and center random intercepts	Adjust effective n/design effect; retain trial-clustered robust inference
Berg 2019	Berg_2019	Executive function	Cluster RCT / Classroom	Clustered design	Yes; mixed models with class and school random intercepts	Cluster correction + core-EF restriction
Chatzihidiroglou 2018	Chatzihidiroglou_2018	Motor competence	Cluster RCT / Classroom	Clustered design	No clear cluster-adjusted analysis	Apply design-effect correction; ICC sensitivity
Chatzipanteli 2007	Chatzipanteli_2007	Motor competence	Cluster RCT / Classroom	Clustered design	No; repeated-measures ANOVA	Apply design-effect correction; ICC sensitivity
Chatzopoulos 2022	Chatzopoulos_2022	Motor competence	Individual RCT / Child	None identified	Not applicable	No cluster correction; dependency handled within trial
Dalala 2024	Dalala_2024	Motor competence	Individual RCT / Child	None identified	Not applicable	No cluster correction
Derri 2001	Derri_2001	Motor competence	Individual RCT / Child	None identified	Not applicable	No cluster correction
Đošić 2025	Dosic_2025	Motor competence	Individual multi-arm RCT / Child, stratified within institution	Shared control	Not applicable	Encode Arm_ID/Control_ID; shared-control covariance
Gencigör 2024	Gencigor_2024	Motor competence	Individual RCT / Child	None identified	Not applicable	No cluster correction; verify any graph-derived SD inputs
Hu 2020	Hu_2020	Motor competence	Randomized trial; unit unclear / Unclear (289 children from 8 classes)	None identified	No; repeated-measures ANOVA	Primary as reported individual allocation; add conservative cluster sensitivity
Intawachirarat 2026	Intawachirarat_2026	Executive function	Individual RCT / Child	None identified	Not applicable	Core-EF model excludes TOL
Jouira 2024	Zinelabidine_Jouira_trial	Motor competence	Individual RCT report / Child	Overlapping report	Not applicable	Same Trial_ID as Zinelabidine; do not double-count participants/trials

Report	Trial ID	Domain	Design / randomization unit	Dependency issue	Source adjustment	Revised analysis handling
Kapodistria 2021	Kapodistria_2021	Motor competence	Individual RCT / Child	None identified	Not applicable	No cluster correction
Liu 2025	Liu_2025	Executive function	Individual stratified RCT / Child within age stratum	None identified	Not applicable	Use Cohort_ID; sampling covariance 0 across disjoint age strata
Oppici 2020	Oppici_2020	Executive function + Motor competence	Hybrid cluster/individual multi-arm RCT / Class first, then child minimization	Shared control; Clustered design	ICC checked: CAMSA 0.002; working memory ~0	Shared-control covariance; use reported near-zero ICC where applicable; sensitivity for other outcomes
Tiktampanidi 2021	Tiktampanidi_2021	Motor competence	Individual RCT / Child	None identified	Not applicable	No cluster correction
Venetsanou 2014	Venetsanou_2014	Motor competence	Individual RCT / Child	None identified	Not applicable	No cluster correction
Williams 2026	Williams_2026	Executive function	Cluster RCT / Kindergarten/classroom	Clustered design	Yes; multilevel with child and kindergarten random intercepts	Use reported design effect 1.9 for primary cluster correction
Zachopoulou 2004	Zachopoulou_2004	Motor competence	Cluster RCT / Classroom	Clustered design	No; repeated-measures MANOVA	Apply design-effect correction; ICC sensitivity
Zhao 2024	Zhao_2024	Motor competence	Parallel individual RCT (paper mislabels as crossover) / Child	None identified	Not applicable	Analyze as parallel RCT; explicitly clarify reviewer concern
Zinelabidine 2022	Zinelabidine_Jouira_trial	Executive function	Individual RCT report / Child	Overlapping report	Not applicable	Same Trial_ID as Jouira; do not double-count participants/trials

Note. Jouira 2024 and Zinelabidine 2022 were linked to one underlying randomized trial and their participants were counted once. Multi-arm trials with shared controls were retained within the sampling variance-covariance structure.

Table S2. Study-specific effect-size conversions and source-data decisions.

Study / effect	Source issue	Primary decision	Sensitivity / documentation
Berg 2019 (EF004–EF013)	Source Table 2 provides adjusted posttest model means with SEs and adjusted regression coefficients; the prior extraction effectively treated model SEs as if they were raw posttest SDs.	Replace Berg effects with standardized adjusted mixed-model coefficients. Four core-EF outcomes remain in the EF primary model; six non-core outcomes remain only in broad-EF sensitivity.	Change-score-only EF sensitivity excludes Berg. Methods will explicitly describe the standardizer and conversion.
Williams 2026 (EF036)	Table 4 labels group values as M(SE), while arm-specific immediate-post SDs are not separately available in the supplied report. The paper analyzes three timepoints with multilevel models and reports overall EF SD=.18 at T1 and .17 at T2.	For the immediate-post primary meta-analysis, calculate the T1–T2 intervention-minus-control change using reported group means and the common overall T1/T2 EF SDs; retain source design effect 1.9.	Sensitivity uses the source longitudinal adjusted contrast (-0.04, 95% CI -0.095 to 0.016) standardized by baseline EF SD, plus Williams-exclusion sensitivity.
Dalala 2024 (MC025 ellipse area)	Source reports intervention pretest mean 7886.46 and posttest 623.51, but the group×time interaction is nonsignificant (F=1.27,p=.26); the value is internally incompatible with the reported analysis and strongly suggests a source typo.	Exclude MC025 from the motor primary model because the baseline value cannot be reliably recovered.	Reinclude the prior likely-typo-corrected extraction as a sensitivity analysis; the motor conclusion is unchanged.
Hu 2020 (MC051–MC062)	Several source Δ entries do not equal the arithmetic pre-to-post change (e.g., gross-motor rows), so the source change table is internally inconsistent.	Use arithmetic pre/post mean differences and reconstruct change SDs from pre/post SDs with r=.50.	Use source-reported Δ dispersion only as a sensitivity analysis; qualitative motor conclusion remains unchanged.
Bentley 2022 (EF001–003, MC010)	The supplied report refers arm-level pre/post statistics to an appendix that is not present, so the pre–post correlation sensitivity cannot be exactly reconstructed for these four effects.	Retain the previously extracted change-score SDs at the primary specification.	Keep Bentley fixed across r sensitivity and run Bentley-exclusion sensitivity.
Gencigör 2024 (MC048–MC050)	Arm-specific change SDs are not reported; change precision was reconstructed from the reported group×time F statistic and verified against the original extraction.	Retain the verified F-derived change precision.	Keep fixed across r sensitivity and run a Gencigör-exclusion sensitivity.
Jouira 2024 + Zinelabidine 2022	The two publications use the same 41 children, same 19/22 allocation, and the same 8-week aerobic-dance trial.	Keep both reports because they contribute different outcome domains, but assign one Trial_ID and count participants only once.	All robust inference clusters at the underlying trial level.

Table S3. Descriptive distribution of primary effect sizes by outcome category.

Domain	Outcome category	Effect sizes	Unique trials	Examples of measures
Executive function	Cognitive flexibility	10	5	Cognitive flexibility; Cognitive flexibility - TMT; Cognitive flexibility - DCCS ACC - 4–5 years
Executive function	Composite EF	1	1	Executive function composite
Executive function	Inhibitory control / executive control	14	6	Inhibition; Inhibition Stroop RT; Inhibition Stroop accuracy
Executive function	Working memory	9	5	Working memory; Working memory - Digit Span; Working memory - BDST - 4–5 years
Motor competence	Balance / postural control	35	10	Balance quotient; Static balance; Dynamic balance
Motor competence	Coordination / rhythm	6	3	Sensorimotor synchronization; Movement reaction time; Sensorimotor synchronization - 80 BPM
Motor competence	Fine / visual-motor competence	10	3	Fine motor quotient; Visual-motor integration; Fine motor precision - drawing lines
Motor competence	Gross / composite motor competence	9	4	General motor age; General motor quotient; Gross motor quotient
Motor competence	Locomotor / mobility	20	4	Locomotor skill - running; Locomotor skill - galloping; Locomotor skill - hopping
Motor competence	Object control / manipulative	16	4	Manipulative skill - striking with bat; Manipulative skill - catching; Manipulative skill - kicking
Motor competence	Other motor competence	7	4	Positive/negative age; Body schema quotient; Spatial organization quotient

Note. This table is descriptive. It reports the distribution of primary effect sizes and trials rather than formal between-subgroup hypothesis tests.

Table S4. Sensitivity analyses for the two primary outcome domains.

Domain	Sensitivity family	Scenario	Effects	Trials	g	95% CI	CR2 p
Executive function	Pre-post correlation	r=0.20	34	7	0.428	-0.033 to 0.889	0.0633
Motor competence	Pre-post correlation	r=0.20	103	17	0.812	0.307 to 1.318	0.0036
Executive function	Pre-post correlation	r=0.50	34	7	0.500	-0.039 to 1.039	0.0634
Motor competence	Pre-post correlation	r=0.50	103	17	0.951	0.386 to 1.516	0.0026
Executive function	Pre-post correlation	r=0.80	34	7	0.687	-0.084 to 1.458	0.0719
Motor competence	Pre-post correlation	r=0.80	103	17	1.300	0.563 to 2.037	0.0018
Executive function	Sampling correlation	rho=0.20	34	7	0.505	-0.033 to 1.043	0.0613
Motor competence	Sampling correlation	rho=0.20	103	17	0.963	0.395 to 1.530	0.0024
Executive function	Sampling correlation	rho=0.50	34	7	0.500	-0.039 to 1.039	0.0634
Motor competence	Sampling correlation	rho=0.50	103	17	0.951	0.386 to 1.516	0.0026
Executive function	Sampling correlation	rho=0.80	34	7	0.496	-0.045 to 1.038	0.0658
Motor competence	Sampling correlation	rho=0.80	103	17	0.939	0.377 to 1.501	0.0027
Executive function	Cluster ICC	ICC=0.01	34	7	0.497	-0.041 to 1.036	0.0643
Motor competence	Cluster ICC	ICC=0.01	103	17	0.958	0.394 to 1.522	0.0024
Executive function	Cluster ICC	ICC=0.05	34	7	0.500	-0.039 to 1.039	0.0634
Motor competence	Cluster ICC	ICC=0.05	103	17	0.951	0.386 to 1.516	0.0026
Executive function	Cluster ICC	ICC=0.10	34	7	0.504	-0.036 to 1.044	0.0625
Motor competence	Cluster ICC	ICC=0.10	103	17	0.944	0.378 to 1.510	0.0027
Executive function	Effect-method / source-data	Change-score-only; exclude Berg adjusted + Williams approximate	29	5	0.629	-0.146 to 1.403	0.0869
Executive function	Effect-method / source-data	Broad EF (all 41 effects; Berg adjusted conversions)	41	7	0.464	-0.061 to 0.989	0.0734

Domain	Sensitivity family	Scenario	Effects	Trials	g	95% CI	CR2 p
Executive function	Effect-method / source-data	Williams source adjusted longitudinal contrast instead of immediate T1-T2 approximation	34	7	0.500	-0.039 to 1.039	0.0637
Executive function	Effect-method / source-data	Exclude Bentley (pre/post SD appendix unavailable)	31	6	0.583	-0.042 to 1.209	0.0617
Executive function	Effect-method / source-data	Exclude Berg adjusted-estimate trial	30	6	0.584	-0.063 to 1.231	0.0677
Motor competence	Effect-method / source-data	Exclude Gencigör F-derived precision effects	100	16	0.781	0.315 to 1.247	0.0028
Motor competence	Effect-method / source-data	Reinclude Dalala ellipse-area effect using current likely typo-corrected extraction	104	17	0.950	0.385 to 1.515	0.0026
Motor competence	Effect-method / source-data	Exclude Bentley (source appendix unavailable for r sensitivity)	102	16	1.004	0.410 to 1.598	0.0026
Motor competence	Source-data	Hu source-reported Δ dispersion	103	17	1.058	0.502 to 1.614	0.0010

Table S5. Result-level RoB 2 assessments used in the revised GRADE evaluation.

Report	Domain	Outcome/result family	D1	D2	D3	D4	D5	Overall
Bentley 2022	Executive function	Core EF: inhibition, working memory, cognitive flexibility	Some concerns	Low risk	Some concerns	Low risk	Low risk	Some concerns
Berg 2019	Executive function	Core EF: inhibition/executive control	Low risk	Low risk	Some concerns	Low risk	Low risk	Some concerns
Intawachirarat 2026	Executive function	Core EF: inhibition, cognitive flexibility, working memory	Some concerns	Low risk	Low risk	Some concerns	Low risk	Some concerns
Liu 2025	Executive function	Core EF across two age strata	Some concerns	Some concerns	Low risk	Low risk	Low risk	Some concerns
Oppici 2020	Executive function	Working memory, cognitive flexibility, inhibitory control	Some concerns	Some concerns	Some concerns	Low risk	Low risk	Some concerns
Williams 2026	Executive function	Executive-function composite at immediate post-intervention	Some concerns	Low risk	Low risk	Low risk	Some concerns	Some concerns
Zinelabidine 2022	Executive function	Inhibition, cognitive flexibility, working memory	Some concerns	Low risk	Low risk	Some concerns	Low risk	Some concerns
Anjos 2018	Motor competence	Motor-development outcomes	Low risk	Low risk	Some concerns	Low risk	Low risk	Some concerns
Bentley 2022	Motor competence	Visual-motor integration	Some concerns	Low risk	Some concerns	Low risk	Low risk	Some concerns
Chatzihidiroglou 2018	Motor competence	Instrumented rhythm/synchronization and movement reaction time	Some concerns	Low risk	Low risk	Low risk	Low risk	Some concerns
Chatzihidiroglou 2018	Motor competence	Observed static-balance performance	Some concerns	Low risk	Low risk	Some concerns	Low risk	Some concerns
Chatzipanteli 2007	Motor competence	TGMD manipulative skills	Some concerns	Some concerns	Some concerns	Some concerns	Low risk	Some concerns
Chatzopoulos 2022	Motor competence	Proprioception, rhythm and balance	Some concerns	Low risk	Low risk	Low risk	Low risk	Some concerns
Dalala 2024	Motor competence	Balance and postural-control outcomes retained in primary meta-analysis	Some concerns	Low risk	Low risk	Low risk	Low risk	Some concerns
Derri 2001	Motor competence	TGMD locomotor skills	Some concerns	Low risk	Low risk	Some concerns	Low risk	Some concerns
Đošić 2025	Motor competence	BOT-2 short-form motor outcomes	Some concerns	High risk	Some concerns	Some concerns	Some concerns	High risk
Gencigör 2024	Motor competence	TGMD-2 object control, locomotor and total scores	Some concerns	Low risk	Some concerns	Some concerns	Low risk	Some concerns
Hu 2020	Motor competence	TGMD-2 gross-motor outcomes	Some concerns	Low risk	Low risk	Some concerns	Low risk	Some concerns
Hu 2020	Motor competence	Instrumented/static and dynamic balance outcomes	Some concerns	Low risk	Low risk	Low risk	Low risk	Some concerns
Jouira 2024	Motor competence	Postural balance (CoP)	Some concerns	Low risk	Low risk	Low risk	Low risk	Some concerns

Report	Domain	Outcome/result family	D1	D2	D3	D4	D5	Overall
Kapodistria 2021	Motor competence	Sensorimotor synchronization/reaction outcomes	Some concerns	Low risk	Low risk	Low risk	Low risk	Some concerns
Oppici 2020	Motor competence	CAMSA motor competence	Some concerns	Some concerns	Some concerns	Low risk	Some concerns	Some concerns
Tiktampanidi 2021	Motor competence	Static, dynamic and functional balance	Some concerns	Low risk	Low risk	Low risk	Low risk	Some concerns
Venetsanou 2014	Motor competence	Rhythmic coordination	Some concerns	Low risk	Low risk	Some concerns	Low risk	Some concerns
Zachopoulou 2004	Motor competence	Jumping and dynamic balance	Some concerns	High risk	High risk	Low risk	Low risk	High risk
Zhao 2024	Motor competence	PGMQ gross-motor domains/subtests	Low risk	Low risk	Low risk	Low risk	Low risk	Low risk

Note. D1 combines the randomization-process judgement with the additional cluster recruitment/identification judgement when applicable, using the less favourable judgement. The target estimand was the effect of assignment to intervention. L = low risk; some concerns and high risk are written in full in the table.
